# A smooth muscle cell lncRNA controls angiogenesis in chronic limb-threatening ischemia through miR-143-3p/HHIP signaling

**DOI:** 10.1172/JCI188559

**Published:** 2025-08-28

**Authors:** Ming Zhai, Anurag Jamaiyar, Jun Qian, Winona W. Wu, Emre Bektik, Vinay Randhawa, Camila Vaz, Arvind K. Pandey, Akm Khyrul Wara, Madhur Sachan, Yi Hu, Jéssica L. Garcia, Claire E. Alford, Terence E. Ryan, Wenhui Peng, Mark W. Feinberg

**Affiliations:** 1 Cardiovascular Division, Department of Medicine, Brigham and Women’s Hospital, Harvard Medical School, Boston, Massachusetts, USA.; 2 Department of Cardiology, Shanghai Tenth People’s Hospital, Tongji University School of Medicine, Shanghai, China.; 3 Department of Cardiology, Tongji Hospital, Tongji University School of Medicine, Shanghai, China.; 4 Department of Applied Physiology and Kinesiology, University of Florida, Gainesville, Florida, USA.

**Keywords:** Angiogenesis, Vascular biology, Cardiovascular disease, Endothelial cells, Noncoding RNAs

## Abstract

Peripheral artery disease (PAD) often advances to chronic limb-threatening ischemia (CLTI), resulting in severe complications such as limb amputation. Despite the potential of therapeutic angiogenesis, the mechanisms of cell-cell communication and transcriptional changes driving PAD are not fully understood. Profiling long noncoding RNAs (lncRNAs) from gastrocnemius muscles of participants with or without CLTI revealed that a vascular smooth muscle cell–enriched (SMC-enriched) lncRNA, CARMN, was reduced with CLTI. This study explored how a SMC lncRNA-miRNA signaling axis regulates angiogenesis in limb ischemia. CARMN-KO mice exhibited reduced capillary density and impaired blood flow recovery and tissue necrosis following limb ischemia. We found that CARMN-KO SMC supernatants inhibited endothelial cell (EC) proliferation, spheroid sprouting, and network formation. RNA-seq identified downregulation of the Hedgehog signaling pathway in CARMN-KO models and revealed that CARMN regulates this pathway through its downstream miRNA, miR-143-3p, which targets Hedgehog-interacting protein (HHIP), an antagonist of Hedgehog signaling. Delivery of HHIP-specific siRNA or miR-143-3p mimics rescued EC angiogenic defects and improved blood flow recovery in both CARMN-KO and WT mice. These findings underscore the critical role of CARMN in modulating angiogenesis through the miR-143-3p-HHIP-Hedgehog signaling axis, providing insights into SMC-EC interactions and potential therapeutic strategies for CLTI.

## Abstract 2

Smooth muscle cell to endothelial cell communication in PAD remains poorly understood. A new lncRNA-miRNA signaling pathway may serve a new approach for PAD therapy.

## Graphical abstract

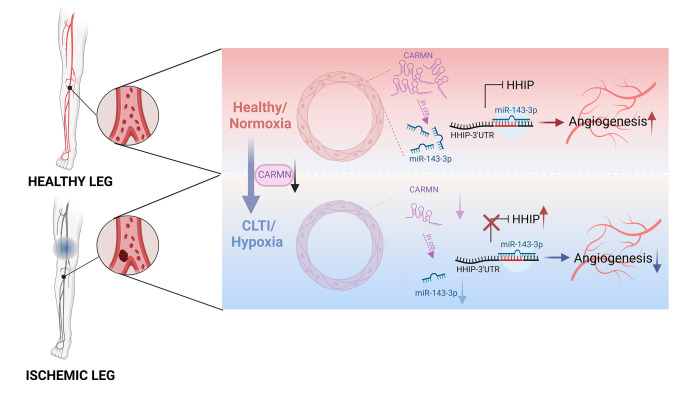

## Introduction

Peripheral artery disease (PAD) involves a gradual narrowing or blockage of arteries in the limbs, reducing blood flow. PAD affects over 200 million people globally (1, 2). Some PAD patients develop chronic limb-threatening ischemia (CLTI), characterized by rest pain, poor wound healing, ulcers, and gangrene, often leading to limb amputation and increased mortality (3). Despite the promise of therapeutic angiogenesis as a potential strategy for treating CLTI over the past several decades, especially in patients without an option for surgery-based therapies, clinical trials exploring angiogenic growth factor therapies have failed to improve perfusion to the ischemic limb in patients with CLTI (4). Accumulating translational studies suggest that this may be partly due to fundamental gaps in understanding how cell-cell communication works and how transcriptional changes in affected individuals lead to cellular and tissue dysfunction (5–7).

Long noncoding RNAs (lncRNAs) refer to RNAs that are longer than 200 nucleotides in length and do not generate proteins (8). Emerging studies over the past few years have shown that lncRNAs can serve as important regulators of angiogenesis (9–12). LncRNAs exhibit divergent roles by interacting with genes or proteins in cis (acting locally) or in trans (acting at a distance), regulating RNA splicing, modulating chromatin architecture, or altering mRNA and protein stability depending on their subcellular localization (13). Although lncRNAs have been implicated in angiogenesis, little is known about their role in cell-cell communication during angiogenesis and in response to ischemia (14). Recently, our group and others identified that the conserved smooth muscle cell–enriched (SMC-enriched) lncRNA CARMN plays a critical role in the development of atherosclerosis by influencing SMC plasticity (15, 16). Reduced levels of CARMN were also linked to abdominal aortic aneurysm (AAA) formation, with its diminution exacerbating AAA formation in mice (17). This growing evidence illustrates that CARMN participates in several vascular diseases. However, the role of lncRNA CARMN in angiogenesis and limb ischemia remains unclear.SMC to endothelial cell (EC) communication may contribute for optimal angiogenic responses in the ischemic limb; however, little is known about this process. We identified reduced expression of the SMC-enriched lncRNA CARMN in patients with CLTI and hypothesized that it may play an important role in angiogenesis and sought to investigate its function in angiogenic studies in vitro or in vivo. Our data show that CARMN-KO mice exhibit reduced capillary density in limbs with impaired blood flow recovery in response to hindlimb ischemia. Because of the lack of expression of lncRNA CARMN in ECs, we explored SMC-EC paracrine effects on a range of angiogenic assays. RNA-seq transcriptomic profiling revealed defective signaling, particularly in the Hedgehog signaling pathway. Further mechanistic studies uncovered a unique miR-143-HHIP signaling pathway that underlies the SMC-EC coupling and angiogenic responses. These studies provide insight into how an SMC-enriched lncRNA can profoundly impact EC angiogenic activity and perfusion after limb ischemia and highlight an important role for cell-cell communication as a target for intervention in therapeutic angiogenesis.

## Results

### CARMN expression is reduced with limb ischemia and deficiency of Carmn-impaired perfusion recovery after hindlimb ischemia in mice.

**Figure 1:**
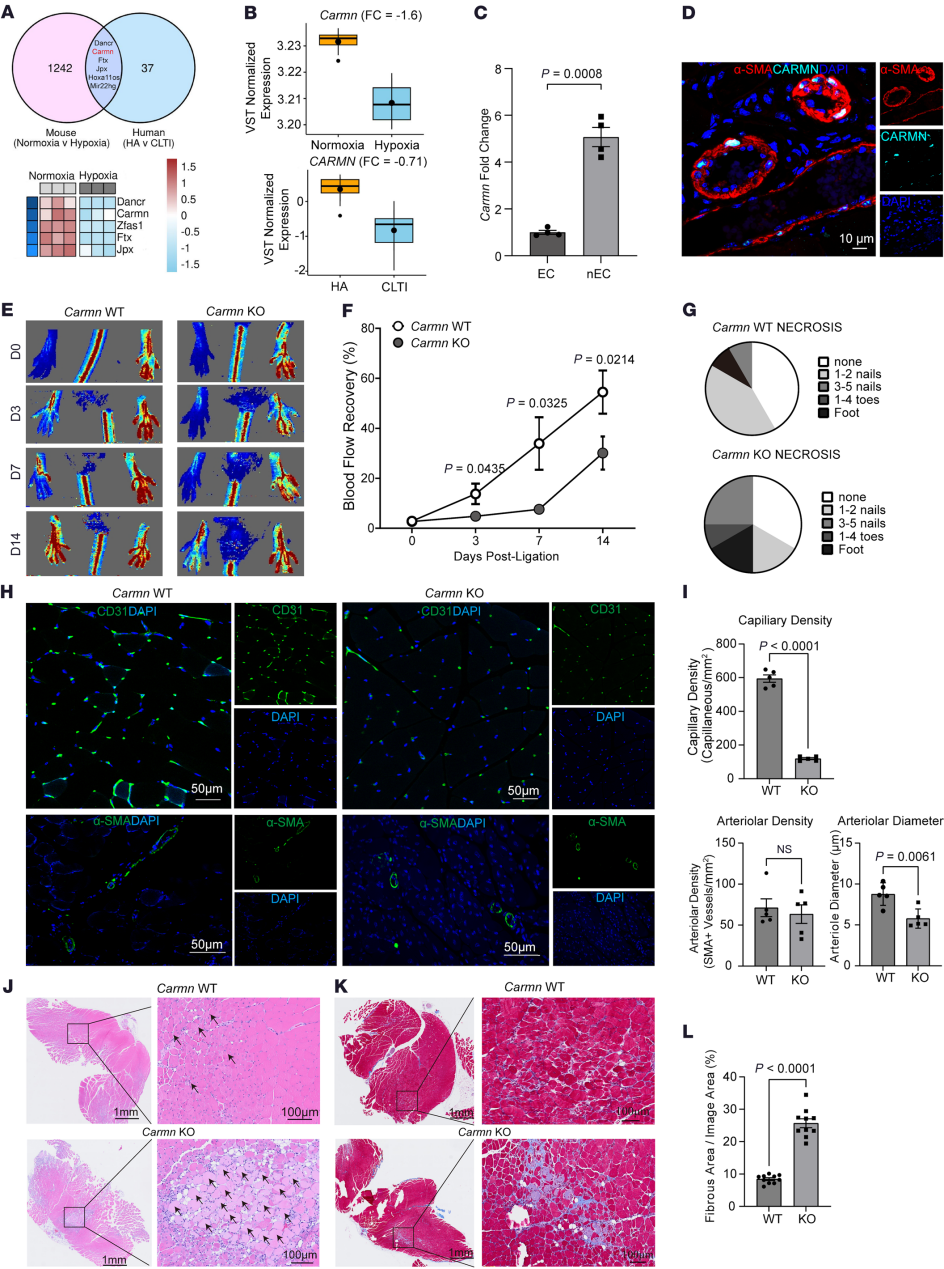
*Carmn* expression is reduced with limb ischemia and deficiency of *Carmn*-impaired perfusion recovery after hindlimb ischemia in mice. (**A**) (*top*) Overlap between differentially expressed lncRNAs screened from hypoxia-stimulated SMCs and differentially expressed lncRNAs screened from human gastrocnemius muscles collected from patients with PAD, healthy adults (HA), or patients with CLTI (GSE120642). (*bottom*) Heatmap of differentially expressed lncRNAs in primary SMCs between normoxia and hypoxia conditions (*P* = 0.00012). (**B**) The expression of *Carmn* in primary SMCs exposed to hypoxia or in gastrocnemius muscles of patients with or without CLTI (*P* = 1.44 × 10^–8^). (**C**) the relative expression of *Carmn* in endothelial cells (ECs) and non-ECs. (**D**) the representative image of *Carmn* colocalized within the nucleus of α-SMA^+^ SMCs within the gastrocnemius muscle harvested from *Carmn* WT mice. (**E**) Representative Laser Doppler Imaging (LDI) images of hindlimbs immediately after FAL and at different time points. (**F**) Quantification of blood flow (surgical / contralateral limb) by LDI images, normalized to the nonsurgery limb between 2 groups, (*n* = 6). (**G**, Necrosis score of ischemic foot 2 weeks after FAL. (**H**) the CD31 immunofluorescence staining in WT and *Carmn*-KO mice. Scale bar: 20 μm. (**I**) the quantification of CD31^+^ capillary density, α-SMA^+^ arteriole density, and diameter of α-SMA^+^ arterioles. (**J**) the representative images of H&E staining of gastrocnemius slides from WT or *Carmn*-KO mice. Scale bar: 100 μm. (**K**) representative images of Masson trichrome staining of gastrocnemius harvested from *Carmn* WT or *Carmn*-KO mice. Scale bar: 100 μm. (**L**) the quantification of the fibrosis areas between groups. For all panels, error bars represent SEM. *P* value was determined by unpaired 2-tailed Student’s *t* test (**B**, **C**, **I**, and **L**) or 1-way ANOVA with Bonferroni post test (**F**).

To identify the presence of lncRNAs in the development of PAD disease, RNA was isolated from the WT SMCs in the presence or absence of hypoxia. Next-generation RNA-seq was conducted to identify differentially expressed lncRNAs in hypoxia-stimulated SMCs. To further capture lncRNAs implicated in PAD, an intersection analysis was performed between differentially expressed lncRNAs screened from hypoxia-stimulated SMCs and differentially expressed transcripts screened from human gastrocnemius muscle tissues collected from patients with CLTI. We found that the *CARMN* was among the top differentially expressed lncRNA that overlapped between these 2 RNA-seq datasets (Figure 1, A and B). Human lncRNA *CARMN* expression has not been investigated in the context of PAD. We evaluated transcriptomic profiling of gastrocnemius muscle RNA from a cohort of patients comprised of healthy adults without PAD, patients with intermittent claudication, and patients with CLTI (18), We found that lncRNA *CARMN* expression was significantly reduced in gastrocnemius muscle from patients with CLTI compared with healthy adults and intermittent claudicants in the GEO online dataset. Furthermore, we also examined *CARMN* expression in an independent cohort by using RT-qPCR in patient gastrocnemius muscle samples from cases with or without CLTI. We similarly found that *CARMN* expression was downregulated in the CLTI group (Supplemental Figure 1A; supplemental material available online with this article; https://doi.org/10.1172/JCI188559DS1). To investigate if *Carmn* expression changed over time in ischemic limbs, we assessed its expression in the gastrocnemius muscle of C57BL/6J mice at days 0, 3, 11, or 31 after femoral artery ligation (FAL). Expression of *Carmn* was markedly reduced by 3 days and remained low over the course of 31 days after FAL (Supplemental Figure 1B). These findings raised the possibility that hypoxia may impact lncRNA *Carmn* expression. Indeed, exposure of primary aortic smooth muscle cells to 24 hours of 2% hypoxia also markedly downregulated *Carmn* expression (Supplemental Figure 1C). To explore the relative enrichment of *Carmn* expression in ECs versus non-ECs, we isolated ECs from the gastrocnemius muscle via CD31 antibody-conjugated magnetic bead pulldown in ischemic limbs of the C57BL/6J mice after 14 days after FAL surgery. The data showed that *Carmn* was mainly expressed in the non-EC fraction (Figure 1C). RNA-FISH also verified that CARMN expression was mainly colocalized within the nucleus of α-SMA^+^ SMC in gastrocnemius muscle (Figure 1D). To determine the role of *Carmn* in experimental limb ischemia, we generated *Carmn*-KO mice. We verified the efficiency of *Carmn* knockout by extracting RNA from gastrocnemius muscle harvested from CARMN^+/+^ or CARMN^–/–^ mice. *Carmn* expression in gastrocnemius muscle from KO mice was significantly suppressed (Supplemental Figure 1D) and was further verified by genotyping (Supplemental Figure 1E). CARMN mainly colocalized within the nucleus in WT α-SMA^+^ SMCs, but it was negligible within *Carmn-*KO α-SMA^+^ SMCs (Supplemental Figure 1F). After FAL surgery, we found that *Carmn*-KO mice had markedly impaired hindlimb blood flow recovery and perfusion (Figure 1, E and F) with higher necrosis scores in the ischemic foot compared with those of WT mice (Figure 1G). Immunostaining of ischemic gastrocnemius muscle demonstrated markedly reduced CD31^+^ capillary density by 80% in *Carmn*-KO mice, whereas there was no difference in SMA^+^ arteriolar density between KO and WT mice. However, the diameter of the SMA^+^ arterioles in *Carmn*-KO mice was moderately smaller compared with *Carmn* WT mice (Figure 1, H and I). After FAL, there was also more severe myocyte necrosis (Figure 1J) and a greater degree of fibrosis in *Carmn*-KO compared with WT mice (Figure 1, K and L). Furthermore, we stained for the pericyte marker NG2 and leukocyte marker CD45 in the gastrocnemius muscles of *Carmn* WT and KO mice after FAL surgery to assess whether *Carmn* affects pericyte and leukocyte recruitment. We found no significant difference in the number of pericytes or leukocytes between the 2 groups. This evidence shows that *Carmn* may not influence the recruitment of pericytes or leukocytes (Supplemental Figure 1, G and H). Our previous study showed that *Carmn* knockdown affected SMC proliferation, migration, and differentiation (15). We therefore explored if *Carmn*-KO SMCs had altered proliferation in vitro after exposure to hypoxia. The data revealed that *Carmn*-KO SMCs have impaired proliferation as quantified by BrdU incorporation (Supplemental Figure 1I), but there was no impact on migration (Supplemental Figure 1, J and K). We also examined the expression of several SMC markers between groups and found that *Carmn*-KO SMCs have reduced expression of *Acta2*, *Cnn1*, *Klf4*, *Sm22a*, and *Myh11*, which are important for SMC development and contractile phenotype (Supplemental Figure 1, L–Q). The loss of *Carmn* may contribute to the decreased diameter of the SMA^+^ arterioles by influencing the expression of these SMC markers. Considering the pivotal role of angiogenesis and microvascular perfusion in restoring blood flow after lower-limb ischemia, we became particularly interested in investigating how genetic deletion of the SMC-enriched *Carmn* could impair endothelial CD31^+^ capillary formation.

### SMC-derived Carmn expression promotes EC proliferation and angiogenesis.

**Figure 2:**
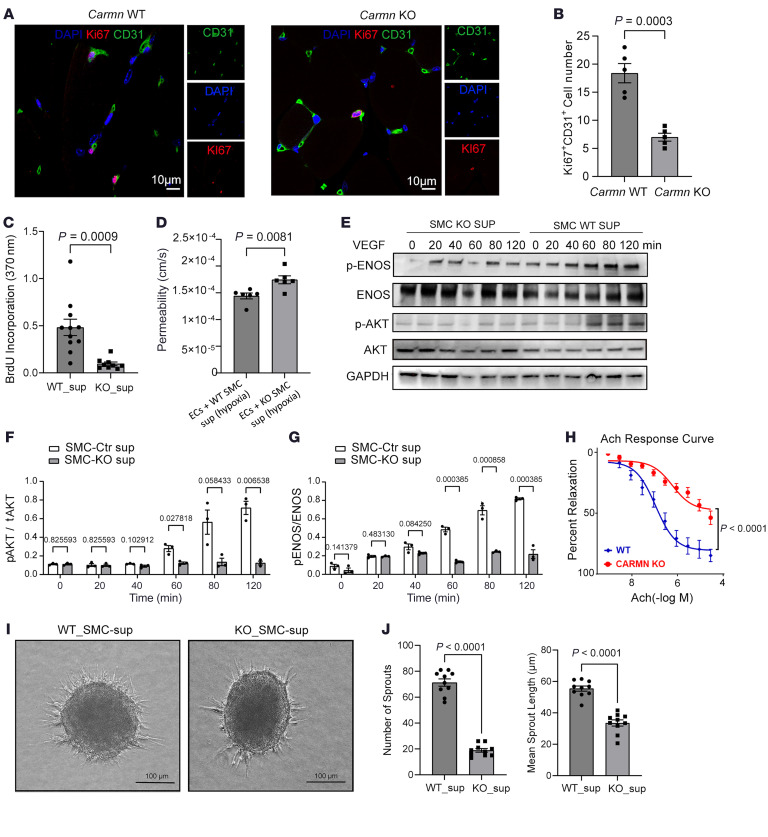
SMC-derived *Carmn* expression promotes EC proliferation and angiogenesis. (**A**) Representative images of CD31^+^ and Ki67^+^ staining between gastrocnemius muscles of WT and CARMN-KO mice. Scale bar = 20 μm. (**B**) the quantification of Ki67^+^ and CD31^+^ cells between groups. (**C**) the quantification of BrdU incorporation of mECs incubated with WT or KO SMC supernatants. (**D**) quantification of permeability assay of ECs incubated with WT or SMC supernatants. (**E**) Representative images of Western blots of the indicated AKT and eNOS proteins in mECs after incubation with WT or KO SMC supernatants. (**F** and **G**) quantification of relative expression of the indicated proteins. (**H**, Ach-mediated arterial vasoreactivity in WT or *Carmn*-KO mice. (**I**) representative images of EC spheroids cocultured with WT or KO SMC supernatants (WT SMC’s supernatants and KO SMC’s supernatants added to the mouse ECs); (**J**) related quantification of spheroid sprout branch length and number; Scale bar: 100 μm. For all panels, error bars represent SEM. *P* value was determined by unpaired 2-tailed Student’s *t* test (**B**–**D**, **F**, **G**, and **J**) or 1-way ANOVA (**H**).

Because *Carmn* is mainly expressed in SMCs and not ECs, yet can influence the angiogenic activity of ECs, we hypothesized that *Carmn* may orchestrate a paracrine-mediated angiogenic mechanism from SMCs to ECs. We first costained CD31 with Ki67 in the gastrocnemius after FAL surgery to assess for EC proliferation. Compared with the WT gastrocnemius group, the number of Ki67^+^ CD31^+^ cells in KO gastrocnemius was reduced by 61.96% (Figure 2, A and B). We collected the supernatants from the cultured WT or KO aortic SMCs and added them to mouse endothelial skeletal muscle cells (mECs) to assess effects on proliferation. Compared with WT SMC supernatant, the KO SMC supernatant suppressed the proliferation of mECs as quantified by BrdU (Figure 2C). To examine the impact of the WT or KO SMC supernatants on EC permeability, we added FITC-labeled dextran to mECs incubated with the supernatants in trans-well chambers. The permeability of mECs incubated with KO SMC supernatants was 20.94% higher than that of mECs treated with WT SMC supernatant (Figure 2D). Furthermore, to assess for changes in markers of EC permeability in vivo, we examined for the protein expression of ZO1, Claudin5, pVE-cadherin-Y658, and VE-cadherin in the gastrocnemius of *Carmn* WT and KO mice after FAL surgery. As shown in Supplemental Figure 2, C and D, CARMN deletion significantly reduced ZO1 and Claudin5 levels, supporting increased vascular permeability. While the ratio of pVE-cadherin-Y658 to total VE-Cadherin was not changed, both markers were significantly reduced individually, suggesting that the weakened junctional function may be due to a reduction in total protein amount rather than a change in its activation state. To explore the impact of SMC-derived supernatants on EC AKT and eNOS signaling, we treated mECs incubated with either SMC-derived WT or KO supernatants to VEGF at different time points. SMC-KO supernatants impeded the activation of pAKT or pENOS (Thr495) in response to VEGF stimulation (Figure 2, E–G). We observed that acetylcholine-induced (Ach-induced) arterial dilation was impaired by up to 39.59% in *Carmn*-KO mice compared with WT mice, indicating impaired endothelium-dependent arterial vasodilation in *Carmn* KO mice (Figures 2H). Because AKT has different isoforms, we examined which isoform might mediate *Carmn*’s action. We found that WT-SMC supernatant activated the expression of pAKT1 instead of pAKT2, whereas *Carmn* KO SMC supernatant reduced the activation of pAKT1. This evidence indicated that *Carmn* WT SMC mainly phosphorylated the pAKT1 isoform. Moreover, we noticed that both pAKT2 and AKT2 expression was lower than pAKT1 and AKT1 expression, respectively. From these findings, we can conclude that AKT1 is the dominant isoform and may mediate CARMN action (Supplemental Figure 2, E and F). Furthermore, we also explored whether SMC *Carmn* influenced the VEGFR signaling pathway or VEGF production in mECs. We found that there was no significant difference in the ratio of pVEGFR/VEGFR of mECs incubated with WT or KO supernatants after being treated with VEGF at different time points (Supplemental Figure 2, G and H). We also found that the WT or KO-SMC supernatants had no significant impact on mEC VEGF production (Supplemental Figure 2I). Furthermore, to explore the impact of the supernatants on angiogenic sprouting, we performed 3-dimensional EC spheroid assays and found that the EC spheroids exposed to WT-SMC supernatants had longer and more sprouts (by 65.40% and 277.24%, respectively) compared with spheroids exposed to KO-SMC supernatants (Figure 2, I and J). Similarly, KO-SMC supernatants also significantly impeded the total count of networks (by 33.25%) in a Matrigel network formation assay (Supplemental Figure 3A). In contrast, there were no significant differences in the apoptotic rate of mECs incubated with WT or KO SMC supernatants (Supplemental Figure 3B). These data showed that the SMC-derived KO supernatants can actively inhibit the proliferation and angiogenic capacity of mECs instead of promoting apoptosis.

### Carmn promotes angiogenic activity by activating the hedgehog signaling pathway.

**Figure 3:**
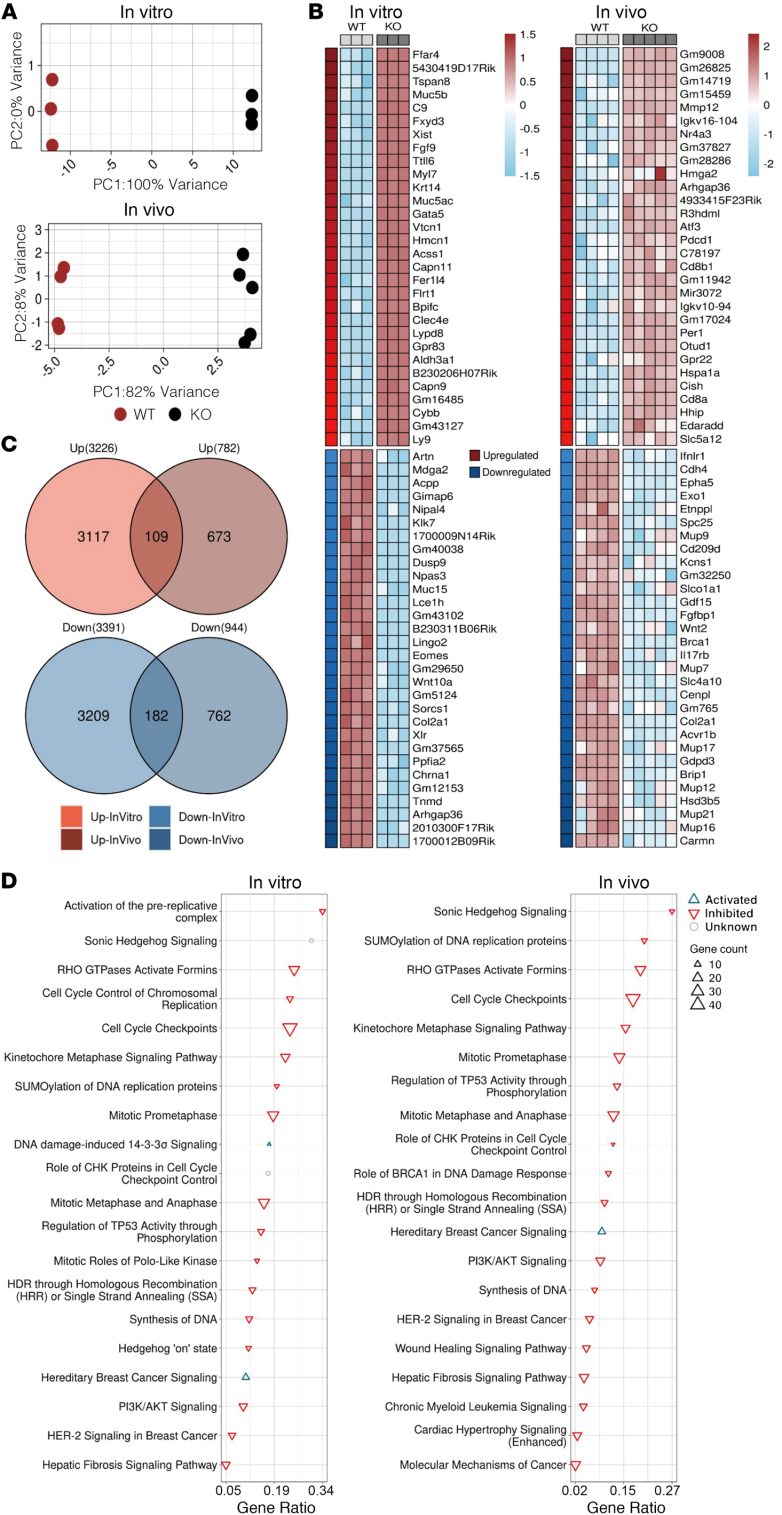
*Carmn* promotes angiogenic activity by activating the hedgehog signaling pathway. (**A**) Scatter plot showing results of a principal component analysis (PCA) of transformed count data from RNA-seq samples. In vitro represents WT or *Carmn* KO SMCs. In vivo represents WT or *Carmn*-KO gastrocnemius muscles after 14 days of FAL. (**B**) Heatmaps of top-30 upregulated (*P* < 0.01 & log_2_FC ≥ 0.58) and top-30 downregulated (*P* < 0.01 & log_2_FC < –0.58) transcripts. The transcripts are prioritized based on their fold-change values, and the upper and lower panels represent upregulated and downregulated transcripts, respectively. (**C**) Venn diagrams representing common upregulated and downregulated transcripts for in vitro and in vivo groups. Red and blue colors represent up- and downregulated transcripts. (**D**) Plot of top-20 significantly enriched pathways (*P* < 0.05). The triangles pointing up are activated (Z > 0), triangles pointing down are inhibited (Z < 0); circles represent pathways with unknown activation status (Z = 0).

**Figure 4:**
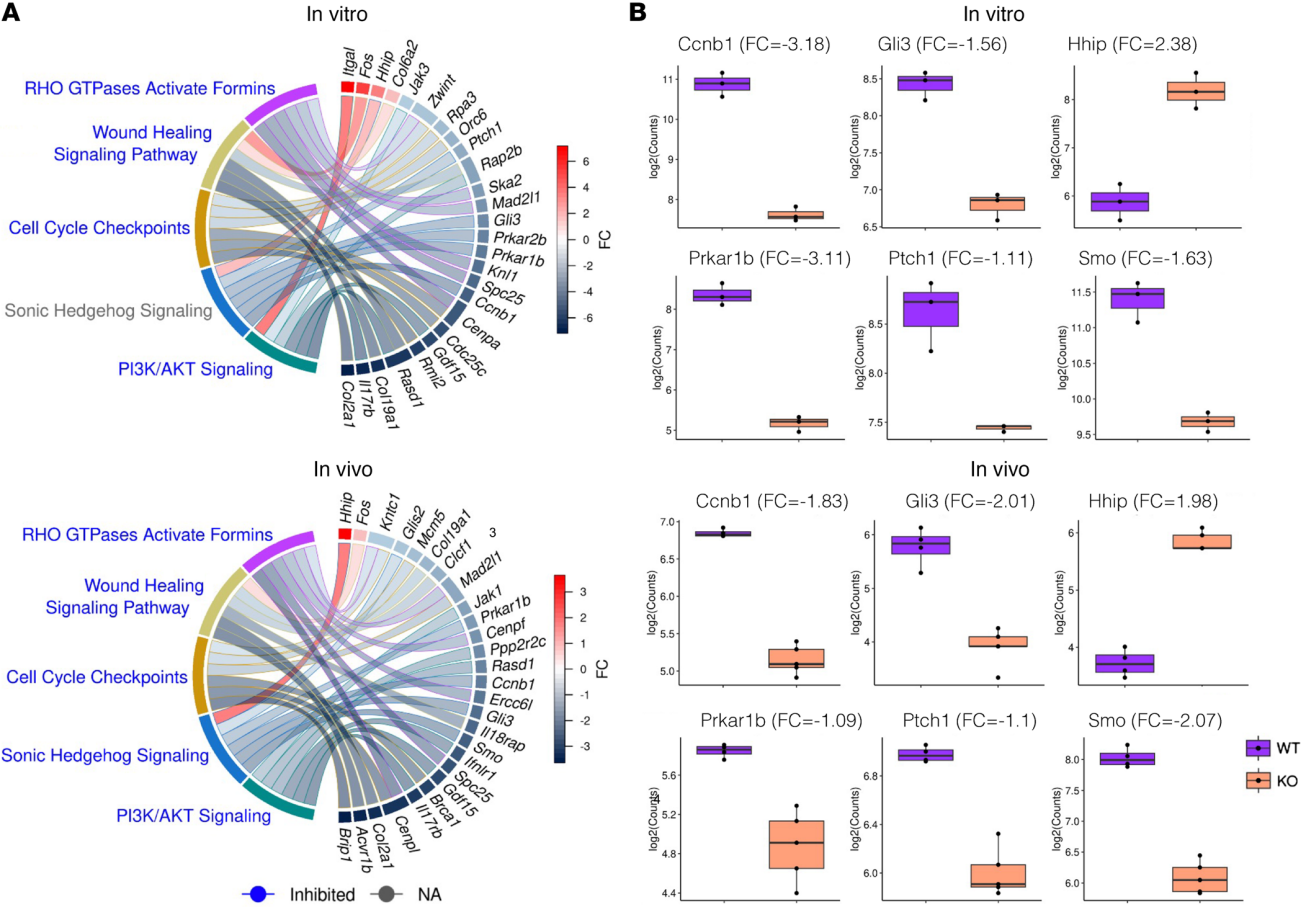
Deficiency of lncRNA CARMN regulates expression of key Hedgehog signaling pathway genes. (**A**) Chordplots of in vitro and in vivo significant (*P* < 0.05) pathways. For each pathway, the top-3 up- or downregulated transcripts (if present) are presented. Blue color–labeled pathways represent inhibited pathways (Z < 0), gray color pathways represent unknown activation status (Z = 0). (**B**) Box plots of expression level between WT and KO in vitro (*top*) and in vivo groups (*bottom*) of well-known representative differentially expressed genes from ‘Sonic Hedgehog Signaling’ pathway. Each boxplot includes the log_2_FC between the 2 groups.

To understand the potential mechanisms by which *Carmn* expressed in SMCs promotes angiogenic activity, we performed RNA-Seq comparing *Carmn* WT and KO aortic SMCs in vitro, and RNA-Seq comparing gastrocnemius muscle from *Carmn* WT and KO mice after FAL surgery. Compared with *Carmn* WT SMCs, 3,228 transcripts were increased and 3,391 transcripts decreased (*P*_adj_ < 0.05). The gastrocnemius of *Carmn*-KO mice after FAL had 782 transcripts increased and 944 decreased compared with the WT mice (Figure 3, A and B). An intersection between upregulated and downregulated transcripts in vitro and in vivo, revealed 109 common upregulated transcripts and 182 common downregulated transcripts from the 2 datasets, respectively. The pathways analysis (Figure 3D) of differentially expressed genes (DEGs) in Figure 3C highlighted several pathways involved in cell cycle such as “Cell Cycle Checkpoints”, “RHO GTPases Activate Formins”, and angiogenesis pathways including “PI3K/AKT signaling” or “Wound Healing Signaling Pathway” that were downregulated in the *Carmn*-KO group both in vitro and in vivo (Figure 3, C and D). Interestingly, the Sonic hedgehog signaling pathway was among the top ranked down-regulated pathways both in vitro and in vivo (Figure 3, C and D). A heatmap analysis of the genes within the Hedgehog Signaling Pathway revealed that they were all downregulated in *Carmn*-KO groups except for the hedgehog-interacting protein (HHIP). *Hhip*, acting as a negative regulator of the Hedgehog signaling pathway, participates in the negative feedback loop of the Hedgehog signaling pathway (19, 20), raising the possibility that *Carmn* may regulate *Hhip* expression (Figure 4A). We observed that key Sonic Hedgehog Signaling pathway genes — including *Ccnb1*, *Gli3*, *Prkar1b*, *Ptch1*, and *Smo* — were downregulated both in vitro and in vivo in CARMN KO compared to wild-type controls, whereas *Hhip* was upregulated (Figure 4B). By analyzing RNA-seq data of siRNA-mediated knockdown of *CARMN* in human coronary artery SMCs (CASMCs), we found similar results of DEGs and enriched signaling pathways (Supplemental Figure 3C)

### Carmn regulates Hhip expression in vitro and in vivo.

**Figure 5:**
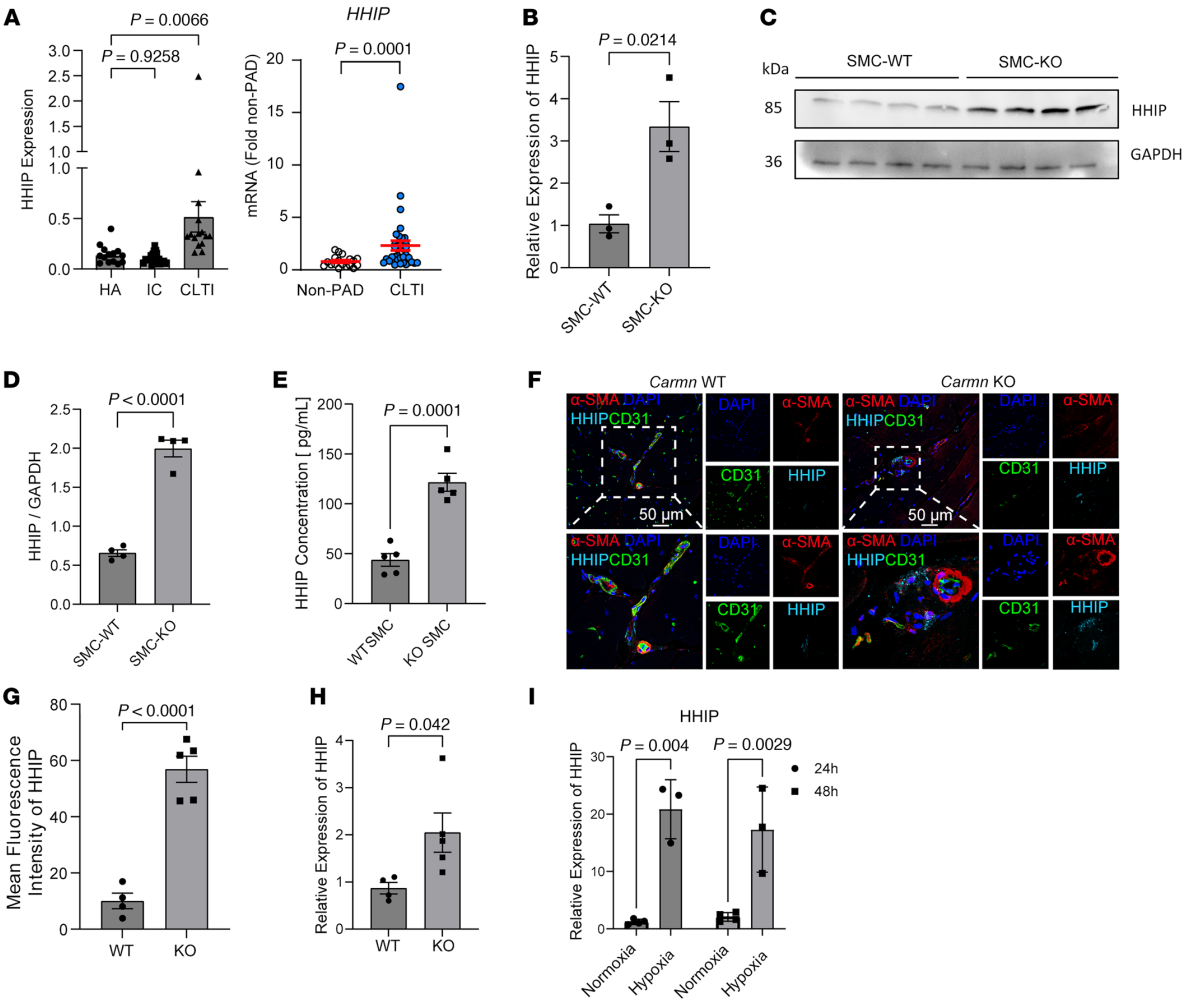
*Carmn* regulates *Hhip* expression in vitro and in vivo. (**A**) Left, Normalized counts of *Hhip* in healthy adults or in patients with intermittent claudication (IC) or CLTI from GEO dataset. (*n* = 13–16). Right, RT-qPCR results of *Hhip* expression in gastrocnemius muscle samples in patients with CLTI and non-PAD control group. (**B**) the relative mRNA expression level between CARMN WT and KO SMCs. (**C**) representative images of Western blots (WB) of *Hhip* between CARMN WT and KO SMCs, (**D**) quantification of WB results in **C**. (**E**) ELISA of HHIP concentration in supernatants collected from WT and KO SMC supernatants. (**F**) the representative images of immunofluorescence for HHIP in gastrocnemius muscles after FAL between *Carmn* WT and KO mice. Scale bar: 20 μm. (**G**) The quantification of the mean fluorescence intensity (MFI) of HHIP. (**H**) the relative expression of *Hhip* in gastrocnemius harvested from 2 groups of mice that underwent FAL surgery. (**I**) The relative expression of *Hhip* in SMC exposed to hypoxia condition after 24 or 48 hours. *P* value was determined by unpaired 2-tailed Student’s *t* test (**A** (right), **B**, **D**, **E**, **G**, and **H**) or 1-way ANOVA with Bonferroni post test (**A** (left) and **I**).

To assess for *HHIP* expression pattern in participants with or without CLTI, we used the same online dataset (GEO) in Figure 1A to assess *HHIP* expression in the gastrocnemius from these patients and found that *HHIP* expression increased in patients with CLTI compared with healthy controls. Furthermore, we also examined the *HHIP* expression by using RT-qPCR in patient gastrocnemius muscle samples collected from cases with or without CLTI. We found that the *HHIP* expression was upregulated in the CLTI group (Figure 5A). To validate our transcriptomic analysis, we also performed a RT-qPCR analysis of *Hhip* expression in *Carmn* WT and KO SMCs and found that *Hhip* mRNA and HHIP protein expression was markedly higher in CARMN-KO SMCs (Figure 5, B, C, and D). To assess if SMCs release HHIP, we performed ELISA for HHIP in the supernatants collected from *Carmn* WT or KO aortic SMCs. HHIP expression was 2.8-fold higher in supernatants collected from *Carmn*-KO SMCs compared with WT SMCs (Figure 5E). Immunofluorescence (IF) staining in the gastrocnemius muscles from *Carmn*-WT or -KO mice after FAL surgery also revealed that HHIP expression was upregulated by 5.65-fold in KO mice compared with WT mice (Figure 5, F and G). Consistent with this, *Hhip* expression in gastrocnemius muscle from the KO mice was higher by 136.13% compared with the WT group after FAL surgery by RT-qPCR (Figure 5H). These data indicated that dynamic changes in *Carmn* expression can regulate *Hhip* expression in vitro and in vivo. To assess if *Hhip* is hypoxia responsive, primary aortic SMCs were exposed to 2% hypoxia. *Hhip* expression in SMCs exposed to hypoxia was 729.90% higher than that in SMCs cultured under normoxia condition (Figure 5I). To further explore if *Carmn* can impact endothelial functional responses, we performed vascular reactivity studies using mesenteric arterioles from *Carmn* WT and KO mice and assayed endothelium-dependent vascular responses.

### Hhip targets the Hedgehog signaling pathway to mediate angiogenic changes in mECs.

**Figure 6:**
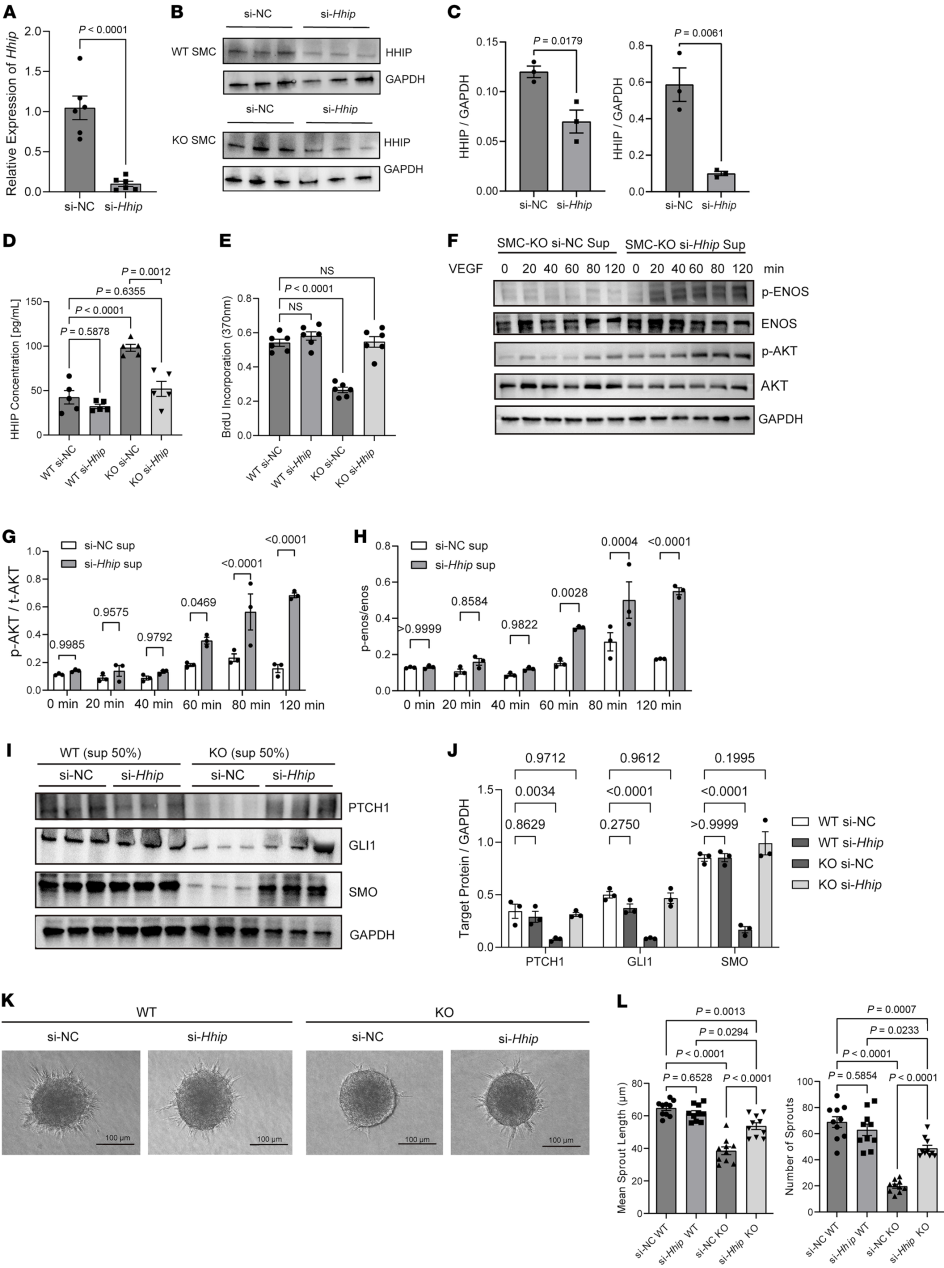
*Hhip* targets Hedgehog Signaling Pathway to mediate angiogenic changes in mECs. (**A**) Relative mRNA expression levels of *Hhip* in KO SMCs with siRNA-mediated (si-mediated) knockdown of *Hhip* or nonspecific control (si-NC) (*n* = 6). (**B**) representative images of the WB of HHIP protein from WT or KO SMC with si-NC and si-*Hhip* transfection. (**C**) the quantification of HHIP protein expression between *Carmn* WT or KO SMCs. (**D**) the HHIP concentration measured by ELISA in supernatants harvested from the indicated groups of SMCs. (**E**) quantification of BrdU incorporation in mECs incubated with supernatants collected from the indicated groups of SMCs. (**F**) the representative WB images of specific AKT and eNOS proteins in mECs incubated with supernatants collected from KO SMCs transfected with si-NC or si-*Hhip*. (**G** and **H**) the quantification of relative expression of p-AKT or p-eNOS of mECs incubated with KO SMC si-NC or si-*Hhip* supernatants. (**I**) representative WB images of the indicated protein expression of the Hedgehog signaling pathway in *Carmn* WT or KO SMCs. (**J**) the quantification of relative expression of proteins in **I**. (**K**) representative images of spheroids cocultured with supernatants collected from the indicated 4 groups of SMCs. Scale bar: 100 μm. (**L**) quantification of branch length and number of sprouts in **K**. For all panels, error bars represent SEM. *P* value was determined by unpaired 2-tailed Student’s *t* test (**A**, **C**, **G**, and **H**) or 1-way ANOVA with Bonferroni post test (**D**, **E**, **J**, and **L**).

The Hedgehog signaling pathway is critical for EC proliferation, migration, and the maturation of newly formed blood vessels implicated in many pathologic conditions, including myocardial ischemia (21–23). The secreted HHIP protein from *Carmn*-KO SMCs might suppress the activity of the Hedgehog signaling pathway in ECs. To address this possibility, we performed siRNA-mediated knockdown studies of *Hhip* in CARMN-KO SMCs and first verified a significant reduction in *Hhip* mRNA transcript and protein expression (Figure 6, A–C). The siRNA-mediated knockdown of *Hhip* in KO SMCs also decreased the HHIP concentration in the collected supernatants as quantified by ELISA (Figure 6D). To assess whether the knockdown of *Hhip* could rescue the inhibitory EC angiogenic effects observed with the supernatants derived from *Carmn*-SMC KOs (Figure 2), we used supernatants with or without *Hhip* knockdown in a similar series of angiogenic assays. The data show that *Hhip* knockdown in SMC-KO supernatants effectively rescued the proliferation of mECs as quantified by BrdU (Figure 6E). To examine effects on AKT and eNOS signaling, we pretreated the mECs with supernatants collected from the *Carmn* SMC KO siRNA-nonspecific control (si-NC) and siRNA-HHIP groups and treated them with VEGF at different time points. Western blot analysis revealed that *Hhip* knockdown robustly increased activation of pENOS or pAKT (Figure 6, F–H). Similarly, the siRNA-mediated knockdown of *Hhip* also significantly accelerated networks in the EC Matrigel network formation assay (Supplemental Figure 3D). In contrast, there was no effect from the supernatants from si-NC and si-*Hhip* of CARMN WT or KO SMCs on apoptosis in mECs (Supplemental Figure 3E). We next assessed the expression of some of the critical proteins in the Hedgehog signaling pathway and found that the knockdown of *Hhip* in KO SMC supernatants strongly rescued the reduction of PTCH1, GLI1, and SMO (Figure 6, I and J). To assess whether expression of mediators of the Hedgehog signaling pathway were altered in patients, we examined the expression for *GLI1*, *SMO*, and *PTCH1* expression between the CLTI and the healthy control groups in the GEO online dataset (GSE12064). The expression of *GLI1*, *SMO*, and *PTCH1* were all downregulated in gastrocnemius muscles in the CLTI group (Supplemental Figure 3F). Furthermore, 3D-spheroid assays of mECs revealed that *Hhip* knockdown in SMC-KO supernatants rescued the antisprouting phenotype of SMC-KO supernatants as quantified by increased sprout length and number of sprouts in mECs (Figure 6, K and L). These data illustrate that the secreted HHIP from SMCs can target the Hedgehog signaling pathway to mediate angiogenic functional changes in mECs.

### Inhibition of the Hedgehog signaling pathway in WT SMCs can phenocopy the antiangiogenetic effects of Carmn-KO SMCs.

**Figure 7:**
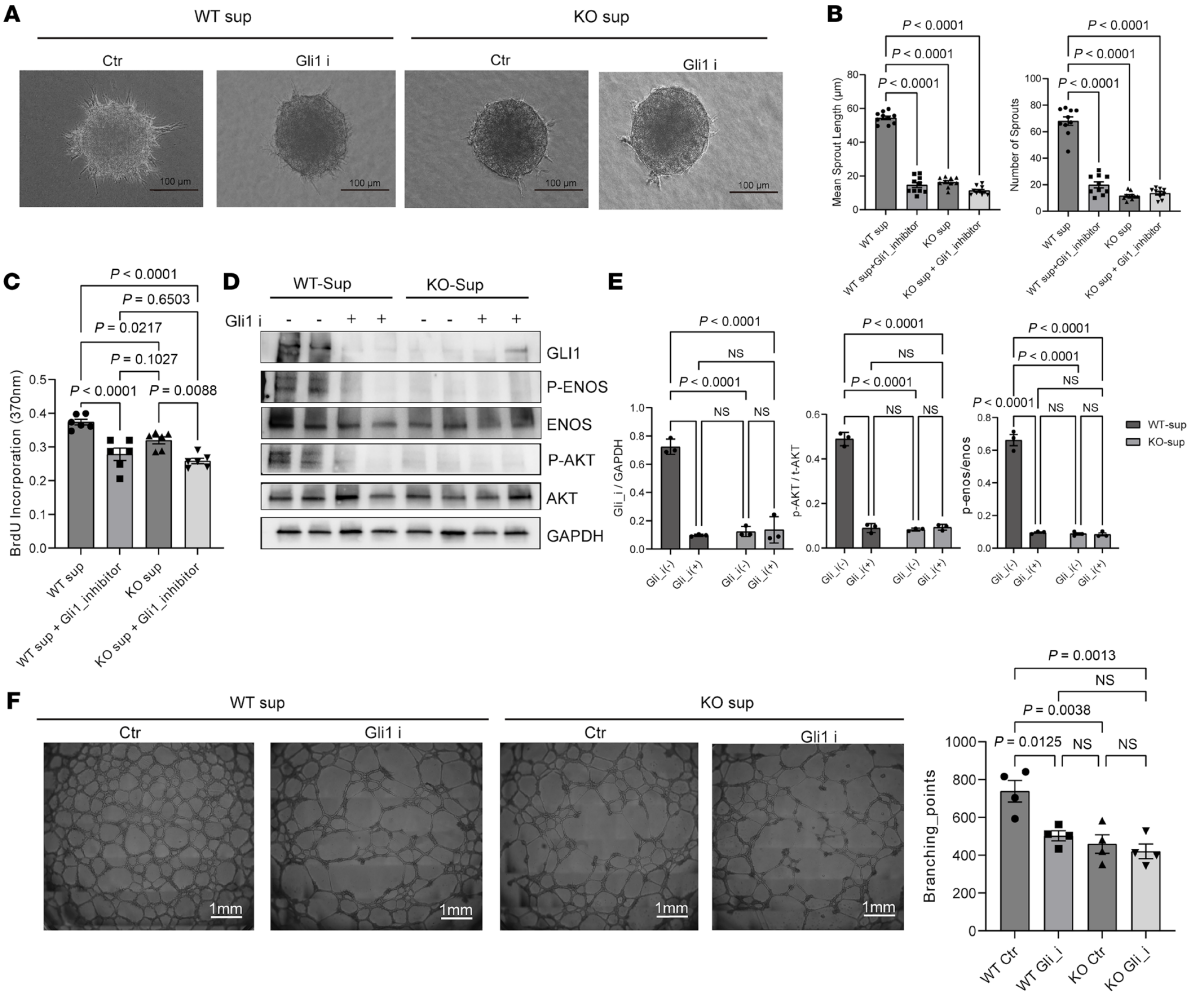
Inhibition of the Hedgehog signaling pathway in WT SMCs can phenocopy the antiangiogenetic effects of *Carmn*-KO SMCs. (**A**) the representative images of mECs spheroid cocultured with supernatants collected from WT or KO SMCs treated with or without Hedgehog signaling pathway inhibitors. Scale bar: 100 μm. (**B**) quantification of spheroid branch length and the number of branches in **A**. (**C**) the BrdU incorporation of mECs incubated with WT or KO SMCs treated with or without Hedgehog signaling pathway inhibitors. (**D**) the representative images of WB results of the indicated AKT or eNOS proteins in mECs after being incubated with supernatants collected from WT or KO SMCs treated with or without Hedgehog signaling pathway inhibitors. (**E**) the quantification of the indicated protein expression levels in **D**. (**F**) representative images of mECs in the network formation assay after incubation with supernatants collected from WT or KO SMCs treated with or without Hedgehog signaling pathway inhibitors. Scale bar: 1,000 μm. For all panels, error bars represent SEM. *P*-value was determined by 1-way ANOVA with Bonferroni post test (**B**, **C**, **E**, and **F**).

To validate the putative role of the Hedgehog signaling pathway in regulating EC angiogenic activity, we used the Hedgehog signaling pathway Gli inhibitor (GANT61) in mECs coincubated with WT or KO-SMC supernatants and performed 3D-spheroid assays. The data show that GANT61 impedes spheroid formation, as manifested by a decrease in the number or length of sprouts, which phenocopied the effects of KO-SMC supernatants on mEC sprouting (Figure 7, A and B). Similarly, GANT61 treatment in WT SMCs also inhibited the proliferation rate of mECs to a similar level exhibited by incubation of KO-SMC supernatants (Figure 7C). Examination of protein expression revealed that the KO-SMC supernatants had markedly reduced expression of GLI1, pENOS, and pAKT similar to the GANT61-treated WT-SMC group. (Figure 7, D and E). Furthermore, mECs were also treated with GANT61 or supernatants for effects on network formation. The data revealed that supernatants from KO SMCs or GANT61 treatment potently inhibited the number of networks of mECs in Matrigel assays (Figure 7F). Taken together, these findings indicate that inhibition of the Hedgehog signaling pathway phenocopies the antiangiogenic effects of supernatants from KO SMCs.

### Carmn inhibits the expression of Hhip through miR-143-3p signaling.

**Figure 8:**
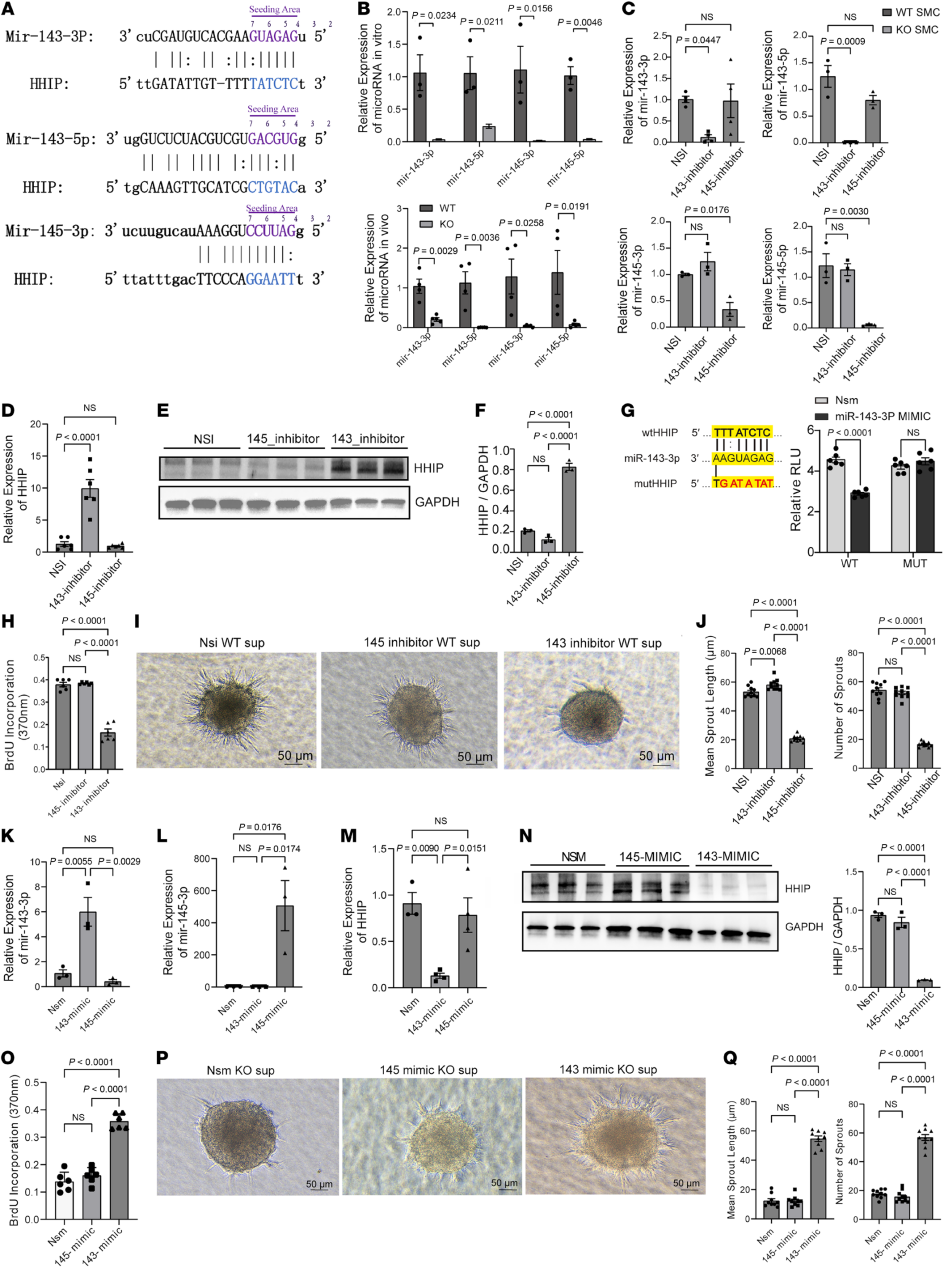
*Carmn* can inhibit the expression of *Hhip* through *miR-143-3p* signaling. (**A**) Schematic of binding sites and sequence complentarity for *miR143* and *miR145* in the *Hhip* 3′ UTR. (**B**) the relative expression of *miR-143-3p*, *miR-143-5p*, *miR-145-3p*, and *miR-145-5p* between *Carmn* WT and KO SMCs in vivo and in vitro. (**C**) the relative expression of *miR-143-3p*, *miR-143-5p*, *miR-145-3p*, and *miR-145-5p* in WT SMC transfected with nonspecific control inhibitor (NSi), *miR143* inhibitor, and *miR145* inhibitor. (**D**) the relative expression of *Hhip* mRNA among WT SMCs transfected with nonspecific control (NSi), *miR143* inhibitor, and *miR145* inhibitor. (**E**) the protein expression of HHIP between WT SMCs transfected with nonspecific control (NSi), *miR143* inhibitor, and *miR145* inhibitor. (**F**) the quantification of relative expression of HHIP among the 3 groups in **E**. (**G**) (Left) Schematic of binding sites between *miR-143-3p* and *Hhip* 3′ UTR. (Right) Relative luciferase units (RLU) of WT *Hhip* 3′ UTR and mutated (MUT) *Hhip* 3′ UTR luciferase reporter assay with NS mimic or *miR-143-3p* mimic (*n* = 6). (**H**) the BrdU assay of mECs incubated with supernatants collected from WT SMCs transfected with nonspecific control, *miR143* inhibitor, and *miR145* inhibitor. (**I**) the representative images of mEC spheroids cocultured with supernatants collected from WT SMCs transfected with nonspecific control (NSi), *miR143* inhibitor, and *miR145* inhibitor. Scale bar: 100 μm. (**J**) the quantification of spheroids branch length and the number of branches of spheroids in **I**. (**K** and **L**) the relative expression of *miR-143-3p* and *miR-145-3p* between groups of KO SMCs transfected with nonspecific control (NSm), *miR-143-3p* mimic, or *miR-143-5p* mimic. (**M**) the relative expression of *Hhip* in KO SMCs transfected with nonspecific control (NSm), *miR-143-3p* mimic, or *miR-143-5p* mimic. (**N**) The WB representative images and related quantification of HHIP in KO SMC transfected with nonspecific control, *miR-143-3p* mimic or *miR-143-5p* mimic. (**O**) BrdU incorporation assay of mECs incubated with supernatants collected from KO SMCs transfected with nonspecific control, *miR-143-3p* mimic, or *miR-143-5p* mimic. (**P**) representative images of mEC spheroid cocultured with supernatants collected from KO SMCs transfected with nonspecific control, *miR-143-3p* mimic, or *miR-143-5p* mimic. Scale bar: 100 μm. (**Q**) quantification of spheroid branch length and the number of branches of spheroid in **P**. For all panels, error bars represent SEM. *P* value was determined by unpaired 2-tailed Student’s *t* test (**B**) or 1-way ANOVA with Bonferroni post test (**C**, **D**, **F**–**H**, **J**–**O**, and **Q**).

We considered several possible mechanisms by which *Carmn* regulates *Hhip* expression, including effects on *Hhip* mRNA stability, potential direct interaction with HHIP protein to regulate its degradation, or by regulating *Hhip* expression through microRNA (miRNA) binding to its 3’ UTR region, given that *Carmn* is the host gene for the *miR143/miR145* cluster. We first explored whether *Carmn* impacts the degradation rate of the *Hhip* mRNA by treatment of WT and KO SMCs with actinomycin D (ActD). There was no significant trend of *Hhip* mRNA expression after ActD treatment between groups (Supplemental Figure 4A), indicating that *Carmn* does not affect the degradation rate of *Hhip* mRNA. Next, we investigated whether CARMN interacts with HHIP protein by performing an RNA immunoprecipitation (RNA-IP) assay. As shown in Supplemental Figure 4B, RTqPCR analysis revealed no significant differences in CARMN expression between the IgG control pulldown and HHIP pulldown samples. To explore whether *miR143* or miR145 regulates the expression of *Hhip* by targeting the 3′ UTR region of the *Hhip* mRNA, we first used prediction algorithms (mirdb.org, freibergtool.org) and identified potential binding sites with complementarity to *miR143* or *miR145* within the 3′ UTR region of *Hhip* mRNA (Figure 8A). Expression of *miR143* and *miR145* decreased in primary KO SMCs or in the gastrocnemius muscle after HLI surgery, respectively, compared with WT controls (Figure 8B). Similarly, the expression of *miR143* and *miR145* were also both significantly downregulated by RTqPCR in gastrocnemius muscles from CLTI patients compared with the non-PAD control group (Supplemental Figure 5, A and B). Interestingly, the expression of *miR-143-3p* was downregulated gradually in the gastrocnemius muscle after surgery, whereas HHIP expression increased (Supplemental Figure 4, C and D). To assess whether *miR143* or *miR145* regulates HHIP expression, we transfected WT SMCs with *miR143* or *miR145* inhibitors (Figures 8C). Because these miRNAs are known to regulate SMC contractile markers (24), we verified that the expression of *Myh11* and *Asma* were successfully downregulated (Supplemental Figure 5, C and D). In contrast, *miR143* inhibition potently increased expression of *Hhip* at the mRNA and protein level (Figure 8, D–F). To verify if *Hhip* is a bona fide target of *miR-143-3P*, we performed luciferase reporter assays with the *Hhip* 3′ UTR. Co-transfection of *miR-143-3p* and WT *Hhip* 3′UTR mediated repression of the luciferase reporter; however, there was no difference observed with *miR-143-3p* and a mutant *Hhip* 3′ UTR containing a scrambled 6 nucleotide change at the seed region (Figure 8G). To explore if the *miR-143*-mediated regulation of *Hhip* in SMCs could impact EC angiogenic activity, we harvested the supernatants from SMCs transfected with the *miR-143* or *miR-145* inhibitors and performed EC angiogenic assays. Supernatant taken from *miR-143*, and not the *miR-145* inhibitor, blocked the proliferation rate of cultivated mECs by BrdU assays and impeded sprout formation in mEC spheroid assays (Figure 8, H–J). If *miR-143* deficiency was mediating the increase in *Hhip* expression in the *Carmn*-KO SMCs, then rescuing its expression might restore its angiogenic paracrine properties. We first transfected *Carmn*-KO SMCs with *miR-143-3p* or *miR-145-3p* mimics and verified upregulated expression of *miR-143-3p* or *miR-145-3p*, respectively. We found that *miR143* overexpression successfully downregulated *Hhip* expression in CARMN-KO SMCs at the mRNA or HHIP protein levels, whereas *miR145* caused no changes (Figure 8, K–M). Consistent with the increase of HHIP protein expression in SMCs mediated by *miR143* inhibition, we also found that the *miR143* inhibitor increased the concentration of HHIP in supernatants of WT SMCs, whereas the *miR-143-3p* mimic decreased the concentration of HHIP in supernatants of KO SMCs (Supplemental Figure 5E). Furthermore, we checked the expression of several Hedgehog genes (*Ptch1*, *Gli1*, and *Smo*) in KO SMCs transfected with non-specific targeted miRNA mimic (NS-m) control and *miR-143-3p*. Expressions of *Ptch1*, *Gli1*, and *Smo* were all rescued in KO SMCs transfected with *miR-143-3p* compared with NS-m control. These results highlight that *miR-143-3p* overexpression within KO SMCs decrease HHIP expression (Figure 8N) and in turn upregulate the expression of the Hedgehog signaling pathway in the SMCs (Supplemental Figure 5F).

To explore if the effects of *miR143* mimics in SMCs could impact EC angiogenic activity, supernatants were harvested and assessed for effects on EC proliferation and spheroid formation. Consistently, only the *miR-143-3p* mimics, and not *miR-145-3p* mimics, increased the proliferation of mECs and increased the branch formation in mEC spheroid assays (Figure 8, O–Q). To assess effects of *miR143* on the AKT and eNOS signaling, we harvested protein lysates of mECs precultured with supernatants collected from WT SMC transfected with non-specific targeted miRNA inhibitor (NSi) control inhibitor and *miR143* inhibitor, and posttreated with VEGF for different time points. Supernatants collected from WT SMC transfected with *miR-143-3p* inhibitors blocked the phosphorylation of AKT or ENOS in mECs (Supplemental Figure 6, A and B). Conversely, we harvested the protein of mECs precultured with supernatants collected from KO SMCs transfected with NS-m control and *miR-143-3p* mimics and posttreated with VEGF for different time points. Supernatants collected from KO SMC transfected with *miR-143-3p* mimics effectively rescued the phosphorylation of AKT or ENOS in mECs (Supplemental Figure 6, C and D).

### Delivery of siRNA targeting Hhip rescued revascularization in hindlimb ischemia.

**Figure 9:**
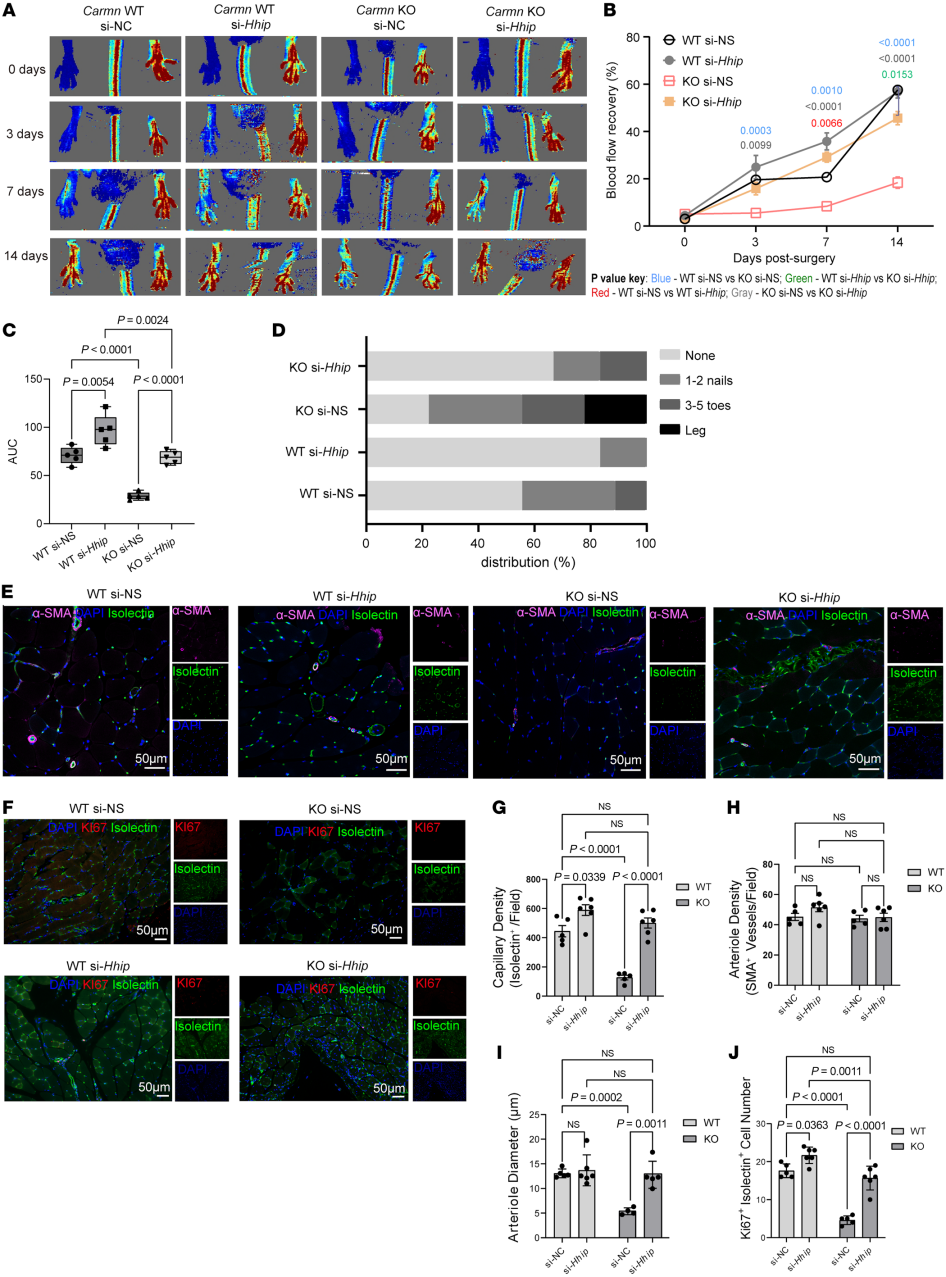
Delivery of siRNA targeting *Hhip* rescued revascularization in hindlimb ischemia. (**A**) Representative Laser Doppler Imaging (LDI) images of *Carmn* WT or KO hindlimbs at different time points after FAL. The indicated mice received 4 intramuscular injections of si-NC or si-*Hhip* over 14 days after FAL. (**B**) Quantification of blood flow (surgical limb/contralateral limb) by LDI images, normalized to the non-FAL limb, (*n* = 6). (**C**) quantification of the AUC between the indicated groups of mice. (**D**) Necrosis score of ischemic foot 2 weeks after FAL. For all panels, error bars represent SEM. (**E**) Confocal micrographs of gastrocnemius muscle immunostained with isolectin^+^ (green) and α-SMA (red). DAPI was used as a nuclear counterstain. (**F**) The immunofluorescence staining of isolectin^+^ (green) and Ki67 (red) under fluorescent microscope. (**G**) The quantification of capillary (isolectin^+^ / Field) density in gastrocnemius muscle in ischemia limb harvested from 4 groups of mice. (**H**) The quantification of arteriole density (α-SMA^+^ / Field) density in gastrocnemius muscle in ischemia limb harvested from 4 groups of mice. (**I**) The quantification of arteriole diameter in gastrocnemius muscle in ischemia limb harvested from 4 groups of mice. (**J**) the quantification of number of KI67^+^ isolectin^+^ endothelial cells in gastrocnemius muscle harvested from the indicated groups of mice. The *P* value was determined by 1-way ANOVA with Bonferroni post tests (**B**, **C**, and **G**–**J**).

Given our in vitro data supporting a *Carmn*-*Hhip* angiogenesis signaling axis, we performed in vivo delivery of *Hhip* or NS siRNAs following FAL in *Carmn* WT or KO mice. The *Hhip* siRNA-mediated knockdown greatly improved blood flow recovery by 44.14% in *Carmn*-KO mice (Figure 9, A–C) at day 14. Even in the WT group, the *Hhip* siRNA delivery also promoted the recovery of the blood flow compared with siRNA control–injected mice. Moreover, *Hhip* siRNA–treated mice experienced a lower frequency of limb necrosis compared with siRNA controls in both *Carmn* WT and KO mice following 2 weeks of FAL (Figure 9D). Immunofluorescence imaging of gastrocnemius muscles at 14 days after FAL surgery revealed that si-*Hhip* injection rescued the capillary density in *Carmn*-KO mice and improved capillary density even in the WT mice (Figure 9, E and G). While the number of arterioles was not different in WT and KO mice after si-*Hhip* injection (Figure 9H), there was a marked increase in arteriolar diameter by 154.61% in the si-*Hhip*–injected KO mice (Figure 9, E and I). In contrast, there was no difference in the arteriolar diameter in si-*Hhip*–injected WT mice. However, endothelial cell proliferation was notably higher in both WT or KO si-*Hhip*–injected mice (Figure 9, F and J). Furthermore, we also verified that the in vivo delivery of si-*Hhip* successfully induced knockdown of *Hhip* in the gastrocnemius muscle in after FAL surgery limb (Supplemental Figure 5, G and H). Taken together, these data indicate that *Carmn* deficiency in SMCs likely contributes to impaired blood flow recovery through increased *Hhip* expression and reduced microvascular endothelial cell proliferation.

### In vivo miR-143-3P delivery improves blood flow recovery in Carmn-KO mice experiencing hindlimb ischemia.

**Figure 10:**
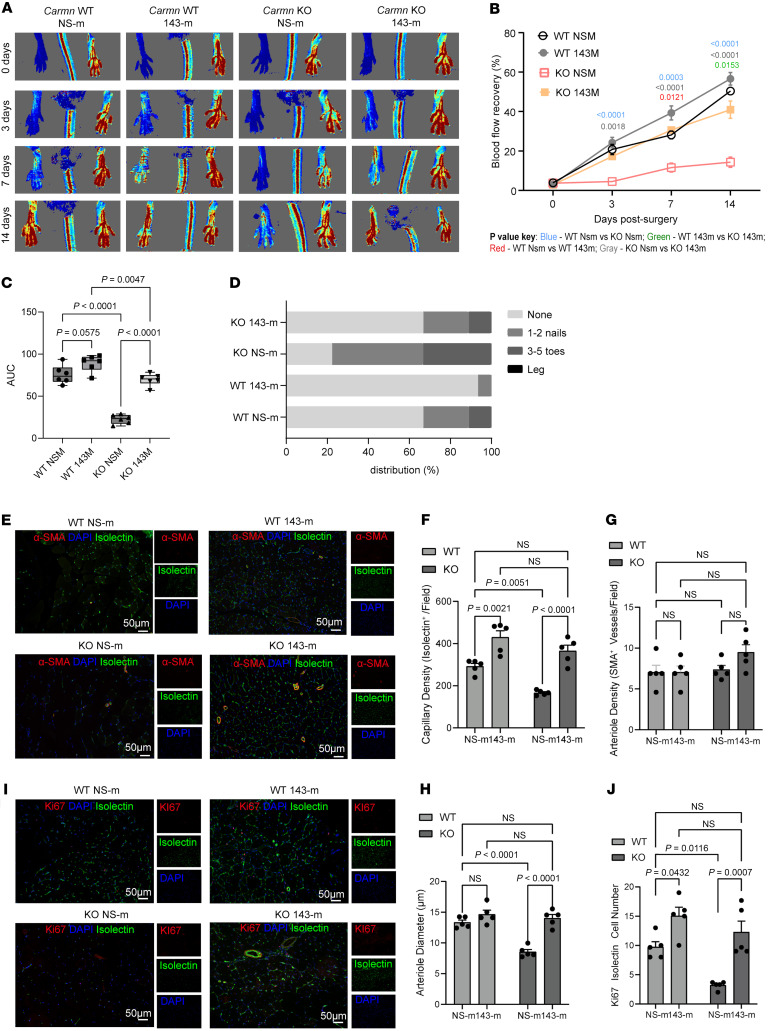
Delivery of *miR-143-3p* in vivo rescued revascularization in hindlimb ischemia. (**A**) Representative Laser Doppler Imaging (LDI) images of *Carmn* WT or KO hindlimbs at different time points after FAL. The indicated mice received 4 intramuscular injections of NS control or *miR-143-3p* mimics over 14 days after FAL. (**B**) Quantification of blood flow (surgical limb/contralateral limb) by LDI images, normalized to the AUC between the indicated groups of mice. (**D**) Necrosis score of ischemic foot 2 weeks after FAL. (**E**) representative IF images of gastrocnemius muscle immunostained with isolectin (green) and α-SMA (red). DAPI was used as a nuclear counterstain. (**F**) the quantification of capillary (isolectin^+^ / Field) density in gastrocnemius muscle in ischemia limb harvested from 4 groups of mice. (**G**) the quantification of arteriole density (α-SMA^+^ / Field) density in gastrocnemius muscle in ischemia limb harvested from 4 groups of mice. (**H**) the quantification of arteriole diameter in gastrocnemius muscle in ischemia limb harvested from 4 groups of mice. (**I**) the immunofluorescence staining of isolectin (green) and Ki67 (red) under fluorescent microscope. (**J**) the quantification of number of KI67^+^ isolectin^+^ endothelial cells in gastrocnemius muscle harvested from the indicated groups of mice. For all panels, error bars represent SEM. The *P* value was determined by 1-way ANOVA with Bonferroni post tests (**B**, **C**, **F**, **G, H**, and **J**).

To evaluate the ability of *miR-143-3p* to rescue the impaired revascularization phenotype in *Carmn*-KO mice, we intramuscularly delivered NS control or *miR-143-3p* mimics into the ischemic gastrocnemius at the time of FAL and over 14 days (Figure 10A). *Carmn*-KO mice treated with *miR-143-3p* mimics exhibited a robust improvement in blood flow recovery by 44.68% (Figure 10, A–C) by day 14. WT mice injected with miR-143-3p mimics also had accelerated blood flow recovery by day 7. Mice treated with miR-143-3p mimics showed robust reductions in necrosis scores compared with mice injected with NS mimic controls (Figure 10D). We also examined the expression of Hedgehog signaling pathway on the protein level in the gastrocnemius muscles harvested from *Carmn-*WT and KO mice after FAL surgery that were treated with NS-m control or miR-143-3p mimics. We found that PTCH1, GLI1, and SMO expression were upregulated in the gastrocnemius muscles from *Carmn*-KO mice treated with *miR-143-3p* mimics compared with those from *Carmn*-KO mice treated with NS-m control (Supplemental Figure 6, E–H). This evidence illustrated that treatment with *miR-143-3p* mimics activated the Hedgehog signaling pathway in vivo. We also verified the efficiency of overexpression of *miR-143-3p* in the limb subjected to FAL surgery. Limbs that underwent FAL surgery and were injected with *miR-143-3p* mimics had higher expression of *miR-143-3p* compared with mice injected with the NS-m control (Supplemental Figure 6I). Immunofluorescence imaging of gastrocnemius muscles at 14 days after FAL surgery revealed that *miR-143-3p* mimic injection rescued the capillary density in *Carmn*-KO mice and improved capillary density even in the WT mice (Figure 10, E and F). While the number of arterioles was not different in WT and KO mice after *miR-143-3p* mimic injection (Figure 10G), there was an increase in arteriolar diameter by 29.16% in the *miR-143-3p* injected–KO mice (Figure 10, E and H). However, endothelial cell proliferation was notably higher in both WT or KO *miR-143-3p*–injected mice (Figure 10, I and J). Furthermore, consistent with our in vitro observations that *miR-143-3p* targets *Hhip*, gastrocnemius muscles of *miR-143-3p* mimic–injected KO mice expressed much lower *Hhip* after FAL compared with KO mice injected with NS mimic controls (Supplemental Figure 6J). In summary, in vivo delivery of *miR-143-3p* reduced target gene *Hhip* expression and rescued blood flow recovery in *Carmn*-KO mice.

## Discussion

Accumulating studies reveal gaps in our understanding of cell-to-cell communication across the spectrum of patients with PAD and its more severe form CLTI (1, 2). Decades of preclinical and clinical research have focused on promoting EC-enriched growth factors to promote vasculogenic processes, including angiogenesis, in an effort to prevent the consequences of severe limb ischemia, including functional impairment, limb necrosis, or limb amputation (25). However, such therapies in patients with CLTI have not been effective in clinical trials and there remains an approximately 20% 1-year incidence of mortality in these patients (26). This study focused on understanding SMC to EC communication by leveraging the SMC-enriched expression of lncRNA *Carmn* and its unexpected paracrine effects on angiogenic activity. In doing so, the findings reveal a novel lncRNA *Carmn*-*miR143*-*Hhip* signaling axis that could be exploited translationally to harness the power of SMC-to-EC–mediated angiogenic activity.

LncRNAs participate in various pathological processes, including angiogenesis, for example, by repressing certain antiangiogenic protein expression or affecting specific signaling pathways on the mRNA or protein levels (27–29). Recently, our group identified *Carmn* as an important conserved lncRNA in SMC plasticity. However, its role in regulating angiogenesis in limb ischemia remained unclear. This study provides evidence of how the SMC-enriched lncRNA *Carmn* can influence the angiogenic response to ischemia by SMCs communicating with ECs. In support, *Carmn* and its target *Hhip* were identified as a key angiogenic regulatory pair in the progression of CLTI through interacting with the Hedgehog signaling pathway. Coculture of mECs with supernatants with elevated expression of *Hhip* collected from *Carmn*-KO SMCs, impaired EC proliferation, spheroid sprouting, and network formation, while siRNA-mediated silencing of HHIP expression rescued this phenotype. Similar phenotypic changes occurred in vivo. In vivo injection of si-HHIP robustly increased blood recovery after FAL surgery and rescued the impaired angiogenesis phenotype observed in *Carmn*-KO mice. The Hedgehog signaling pathway regulates a wide range of developmental processes, including vasculogenic processes, and angiogenesis by secreting proangiogenic factors (30, 31). *Hhip* is known to compete with *Shh* at the receptor of the Hedgehog signaling pathway, thereby serving as an antagonist of the pathway (19, 20). For example, *Hhip* may confer antitumor properties by inhibiting angiogenesis and EC proliferation during tumor development (32, 33). However, few studies have investigated the role of *Hhip* in regulating the angiogenic properties in limb ischemia or wound recovery in response to injury. In humans, *CARMN*, *HHIP*, and members of the hedgehog signaling pathway (*GLI1*, *PTCH1*, and *SMO*) exhibit expression patterns that mirror results from mouse in vivo studies. *CARMN* expression was significantly reduced in the gastrocnemius muscles of IC and CLTI patients compared to healthy adults, while *HHIP* expression was considerably higher in the CLTI group. *CARMN* mRNA was also significantly downregulated in a separate cohort (non-PAD versus CLTI), while *HHIP* transcripts were overabundant. Expression of *miR-143-3p* and *miR-145-3p* was also suppressed in the CLTI group compared with the non-PAD group. The agreement in the expression pattern for this pathway between the 2 human cohorts and the mouse in vivo data highlights the translational relevance of *Carmn*-*miR-143*-3p-*Hhip* signaling axis in the context of PAD.Intriguingly, our data revealed a pronounced decrease in *Carmn* expression during the angiogenic phase of HLI recovery that corresponded with an increase in *Hhip* expression. As a result, we considered that *Carmn* may exhibit proangiogenic effects. This premise was supported by our RNA-seq data in vitro and *Carmn*-KO mice in vivo that showed several proangiogenetic signaling pathways, including the PI3K-AKT signaling pathway, wound healing signaling pathway, and cell cycle checkpoints, were all inhibited. Consistent with this finding, there was a decrease in both capillary density and arterial diameter of vessels in the gastrocnemius muscles of *Carmn*-KO mice after FAL surgery compared with WT mice. These data indicated that the *Carmn* may facilitate vessel growth and angiogenesis by regulating the Hedgehog signaling pathway.

Angiogenesis and arterialization require a complicated series of cell activations, including from neighboring perivascular cells such as SMCs that are often in proximity with the endothelium of capillaries or of medium-to-larger sized vessels (34, 35). The indirect paracrine mechanisms between ECs and SMCs can influence EC differentiation, migration, and proliferation (36). Our study found a critical role of SMCs in promoting the angiogenic activity of ECs. The data support that under homeostatic nonischemic conditions, *Carmn* suppresses the expression of *Hhip*, which has antiangiogenic properties. Within the context of the ischemic gastrocnemius muscle in patients with CLTI or in mice after FAL induction, the expression of *Carmn* was downregulated, whereas the expression of *Hhip* was upregulated. Consistent with this inverse relationship, supernatants containing high levels of *Hhip* from *Carmn*-KO SMCs impaired EC spheroid sprouting, network formation, and proliferation, thus highlighting the antiangiogenic function of *Hhip*. The application of si-*Hhip* within *Carmn*-KO SMCs rescued this phenotype, indicating coupling of this lncRNA-protein pair. Interestingly, there were no detectable differences for the recruitment to vessels for NG2^+^ pericytes or CD45^+^ leukocytes in *Carmn*-KO or WT mice after HLI. These findings further strengthen the premise that SMCs promote angiogenic induction likely through a paracrine mechanism. Several studies demonstrated that *Hhip* can be secreted into the tissue environment and that it plays an essential role in the regulation of processes, such as tumorigenesis and adipogenesis, by interacting with the Hedgehog signaling pathway (19, 37). Our findings build upon other studies that have shown the importance of cooperativity of SMCs and ECs for maximizing tissue perfusion and repair. For example, coadministration of iPSC-derived SMCs and ECs after hindlimb ischemia in mice increased limb salvage in ischemic limbs compared with transplantation of either alone (38). This study ascribes a role for *Hhip* function in regulating EC angiogenic properties in limb ischemia and uncovers how lncRNA *Carmn* is linked to regulating *Hhip* via a miRNA intermediary.

Several lines of evidence support that deficiency of lncRNA *Carmn* upregulates HHIP via the intermediary of *miR-143-3p*. First, *Carmn* serves as a host gene and is located in proximity to *miR-143-3p*, an effect that can promote *miR-143-3p* expression (39, 40). Inhibition of *miR-143-3p* suppressed the ability of *Carmn* to inhibit the expression of *Hhip* in WT SMCs. Consistent with an inverse relationship of *miR-143* and *Hhip* observed in SMCs in our studies, this relationship was also observed when we interrogated a curated RNA-seq dataset of SMC-enriched ureters harvested from the *miR143*/*miR145*-KO and control mice, which showed that *Hhip* expression was upregulated and the Hedgehog signaling pathway was inhibited (41). In our study, the reduced complementarity of the *miR145* seed sequence in the 3’ UTR of *Hhip* (compared with the *miR143* seed sequence, Figure 8A) likely explains its lack of effects on *Hhip* expression or in EC angiogenic experiments cocultured with SMC supernatants. Both *miR-143-3p* overexpression and siRNA-mediated knockdown of *Hhip* decreased the expression of *Hhip* in vivo, and rescued the impaired blood flow recovery, angiogenesis, and tissue necrosis phenotype observed in *Carmn*-KO mice. Thus, the relationship between host gene *Carmn* and *miR-143-3p*, and their cooperative function in regulating angiogenesis after limb ischemia offers insights into understanding the role of miRNAs and their host genes in this process.

In conclusion, the SMC-enriched lncRNA *Carmn* potently regulates the EC angiogenic response to limb ischemia. Gastrocnemius muscles of patients with CLTI exhibit lower CARMN and higher HHIP, consistent with an antiangiogenic state. Genetic loss of *Carmn* exhibited reduced blood flow recovery, decreased capillary density and smaller diameter vessels, and higher tissue limb necrosis after FAL surgery. Our studies in vitro and in vivo demonstrated that *Carmn* suppressed *Hhip* expression or secretion via regulation of *miR-143-3p* that directly binds the 3’ UTR region of *Hhip*. Functional assays and rescue experiments verified that the *Hhip* knockdown or *miR-143-3p* overexpression rescued the phenotype of impaired blood flow recovery, angiogenesis, and limb necrosis in both *Carmn*-KO and WT ischemic limbs. Collectively, these findings support an important SMC-to-EC paracrine role in regulating angiogenic responses in ischemic limbs and provide targets for intervention by regulating the lncRNA *Carmn*-*miR143*-*Hhip* signaling axis.

## Methods

### Sex as a biological variable.

Out study examined male and female animals, and similar findings were reported for both sexes.

Additional details on methods can be found in the Supplemental Methods.

### Human study.

Two different cohorts were examined in this study. The first cohort for lncRNA profiling examined the RNAseq dataset (GSE120642) involving gastrocnemius biopsies from healthy adults (HA), patients with intermittent claudication (IC), or patients with CLTI. The patient characteristics for this dataset are previously described (42). The second independent cohort used for RTqPCR in this study was a cross-sectional study involving gastrocnemius biopsies from participants without PAD or those with CLTI. Participants were recruited through the University of Florida Health and the Malcom Randall VA Medical Centers in Gainesville, Florida, USA. Patients with a normal ABI (greater than 0.9) or those with an abnormal ABI (less than 0.9) indicating a diagnosis of PAD with a Rutherford Stage between 4–6 set as inclusion criteria. Patients with nonatherosclerotic occlusive disease were excluded (acute limb ischemia, vasculitis, Buerger’s disease, etc.). Medical history, race, smoking history, and demographics were obtained by self report. Coexisting medical conditions and medication usage was obtained through review of medical records. Individuals who were non-PAD controls were recruited within the University of Florida Hospital System and Malcom Randall VA medical center and were free from peripheral vascular disease. Gastrocnemius muscle specimens were collected via percutaneous muscle biopsy using sterile procedures as previously described. This procedure involved a small (< 0.5cm) incision through the skin and placement of a suction-controlled sterile 14-gauge biopsy needle (BD Elevation Breast Biopsy System) into the gastrocnemius muscle. Typical yield from this approach ranges from 80–300 mg of muscle tissue. Total RNA was extracted from gastrocnemius muscle using the Direct-zol RNA MiniPrep kit (Zymo Research, Cat. No. R2052) following the manufacturer’s direction. The related patient physical characteristics were submitted as Supplemental Table 1.

### Animal studies.

Studies were performed in CARMN^+/+^ and CARMN^–/–^ mice (The Jackson Laboratory). All male and female mice used were age-matched in experiments and maintained under SPF conditions at an American Association for the Accreditation of Laboratory Animal Care accredited animal facility at the Brigham and Women’s Hospital.

### Hindlimb ischemia mouse models.

Mice were subjected to FAL surgeries to replicate critical limb ischemia as we have described (43–48). Briefly, mice were injected i.p. with 150 μl of 20% ketamine/5% xylazine in 0.9% saline. Once anesthetized, the right medial thigh to the suprapubic area was treated with a commercial emollient to remove fur and sterilized with Povidone iodine. Skin and fascia were dissected away to reveal the femoral bed. The femoral artery and surrounding tissue were proximally and distally ligated with 7-0 Prolene sutures. The arterial bed in between sutures was cauterized. Abrogation of blood flow compared with the contralateral limb (< 10%) was confirmed using a laser Doppler imager (Moor Instruments, UK). Percent blood flow recovery was calculated by comparing a ratio of ischemic paw to contralateral paw Doppler count profiles and normalized blood flow recovery was calculated by comparing the ratio of ischemic to contralateral paw Doppler count profiles to day 0 postoperative percent blood flow. Necrosis scores were calculated as the percentage of mice in a group that fall under one of the following limb necrosis categories on day 14 after surgery: (a) none (no necrosis); (b) 1–2 nails lost; (c) 3–5 nails lost; (d) 1–4 toes lost; (e) whole foot lost.

### Cell culture and transfection.

Mouse primary endothelial cells (mECs) were cultured in endothelial cell growth medium (Cell Biologics, M1168). Cells that were utilized for experiments were passaged no more than 6 times. MOVAS cells (ATCC, CRL-2299) and Primary smooth muscle cells (SMCs) were cultured in SMC media (DMEM Medium (DMEM; Gibco, 10566-016) supplemented with 10% fetal bovine serum (FBS) and 1% Penicillin-streptomycin (P/S)). Transfection was performed using Lipofectamine 2000 (Invitrogen) as described in the manufacturer’s protocol. Negative control inhibitor (4464076), miR-143-3p inhibitor (5’-UGAGAUGAAGCACUGUAGCUC-3’; #MH10883, 4464084), miR-145-3p inhibitor (5’-GGAUUCCUGGAAAUACUGUUCU-3’; #MH13036, 4464084), negative control mimic (4464058), miR-143-3p mimic (sequence 5’-UGAGAUGAAGCACUGUAGCUC-3’; #MC10883, 4464066), miR-145-5p mimic (5’-GUCCAGUUUUCCCAGGAAUCCCU-3’; #MC11480, 4464066), are all from ThermoFisher Scientific and used for transfection at 100 nM in primary SMCs. SMCs were transfected with negative control siRNA (The sequences were UGGUUUACAUGUCGACUAA, Dharmacon, 3391201) or HHIP siRNA (GUAGGGUUUUGAAAUGUUC, Dharmacon, 240617) with Lipofectamine 2000 at 100 nM.

### RNA Isolation and real-time quantitative PCR.

Total RNA was extracted by using TRIzol reagent following the manufacturer’s protocol (Invitrogen, 15596-026). The concentration and quality control of RNA was examined using NanoDrop 2000 (ThermoFisher). miRNAs were reverse transcribed using miRCURY LNA RT Kit (339340) according to the manufacturer’s instructions. MiRCURY LNA SYBR Green PCR Kit (Qiagen, 339346) was used for quantitative real-time PCR analysis with the Quantstudio 6 Pro (ThermoFisher) following the manufacturer’s instructions. miR-143-3p (Qiagen, YP00205992), miR-143-5p (Qiagen, YP02109892), miR-145-3p (Qiagen, YP00204192), and miR-145-5p (Qiagen, YP00204483) expression levels were normalized to U6 snRNA (Qiagen, YP02119464) and were calculated using 2-DCt method. cDNA for mRNA was produced using High-Capacity cDNA Reverse Transcription Kit (ThermoFisher, 4368814). mRNA expression levels were normalized to GAPDH and were calculated using 2-DCt method. Subsequent RT-qPCR was performed using GoTaq qPCR Master Mix (Promega, 0000599886). The list of primers is provided in Supplemental Table 2.

### Western blot analyses.

Cells were lysed in RIPA buffer (ThermoFisher Scientific, USA) containing 1% protease and phosphatase inhibitors and resolved by SDS-PAGE. The proteins were separated by gel electrophoresis and then transferred onto PVDF membranes (Bio-Rad, USA). The membranes were blocked with 5% FBS in 1X TBST at room temperature for 1 h and incubated overnight at 4°C with antibodies against HHIP (Proteintech, 29466-1-AP, 1:1000), GAPDH (Cell Signaling Technology, D16H11, 1:5000), PTCH1 (Santa Cruz,sc-518102, 1:1000), SMO (Santa Cruz,sc-166685, 1:1000), Gli1 (Proteintech, 66905-1-Ig, 1:1000), P-AKT (Cell Signaling Technology,4060S, 1:1000), AKT (Cell Signaling Technology, 4691S, 1:1000), P-ENOS (Thr495) (Proteintech, 28939-1-AP, 1:1000), or ENOS (Cell Signaling Technology, 32027S, 1:1000). Membranes were incubated with secondary antibody (anti-rabbit IgG, HRP-linked Antibody [Cell Signaling Technology, #7074]) for 1 h at room temperature. Protein bands were detected by enzyme-linked chemiluminescence using a luminescent image analyzer (Bio-Rad, Chemidoc).

### Native RNA immunoprecipitation assay.

Primary mouse SMCs or MOVAS cells were used for immunoprecipitation of HHIP-bound RNAs as previously described (49). Human CASMCs were used as a positive control to validate previously identified interaction of SRF and CARMN (15). Briefly, SMCs were seeded in large flasks to yield at least 10 million cells at approximately 80% confluency per sample, harvested by trypsin digestion, washed with PBS and lysed in ice-cold 1X PLB buffer supplemented with 1mM dithiothreitol (DTT) (ThermoFisher, Cat: P2325), 200 units/ml RNase OUT (ThermoFisher, Cat: 10777019), and EDTA-free Protease Inhibitor Cocktail (ThermoFisher, Cat: PI78441). Cells were frozen at –80°C for at least a day to improve cell lysis. Antibodies for HHIP, SRF (CST, 5147) or IgG isotype control antibodies (Millipore, PP64B) were conjugated to Pierce Protein A/G magnetic beads (ThermoFisher, Cat: 88802), and total protein samples from cell lysates were incubated with beads overnight at 4°C in 1X NT2 buffer (supplemented with 20mM EDTA pH 8.0, 1mM DTT, and 200U/ml RNase OUT) and washed with 1X NT2 buffer to remove unbound proteins. Captured protein-bound RNAs were eluted by Proteinase digestion and reverse transcribed for qPCR quantification. Gene expression values were normalized to 10% input RNA.

### RNA-Seq Bioinformatic analysis.

RNA-Seq transcriptomic analysis was performed after ribodepletion and library construction by using Illumina HiSeq2500 V4 2 × 150 PE (Genewiz). For in vitro and in vivo datasets, the obtained reads were examined for quality control using FastQC v0.12.1(www.bioinformatics.babraham.ac.uk/projects/fastqc). The reads were trimmed to remove possible adapter sequences and nucleotides with poor quality using Trimmomatic v0.36 (50). The trimmed reads were then mapped to the Mus musculus GRCm38 reference genome (www.ensembl.org) using the STAR aligner v.2.5.2b (51). For in vitro and in vivo datasets, the mean quality scores of all samples were 37.60 and 38.45, respectively, which indicated all samples to be of high quality, and the mean of total reads per sample was 19,125,123 and 21,326,915, respectively. Subread v1.5.2 (52) was used to compute unique gene hit counts. The gene hit counts table was used for further downstream differential expression analysis. Quality assessment of the samples was also performed by clustering them based on the gene expression profiles and then visualizing the samples in the 2D plane spanned by their first two principal components (i.e., PC1 and PC2). In both the groups, WT and KO states were separated from each other along dimension 1 (the x-axis) and also indicating absence of any outlier sample. The in vitro group comprised 3 samples each for WT and KO state while the in vivo group comprised 4 samples for WT and 5 samples for KO. Before the differential expression analysis, the transcripts with a count of at least 5 for a minimal number of samples were considered. Using DESeq2 v1.42.1 (53), a comparison of gene expression between states WT (control) and KO (subject) was performed both for in vitro and in vivo groups. Wald test was used for hypothesis testing during comparing WT and KO groups, and the transcripts with *P* value < 0.05 and log_2_ fold change of 0.58 were considered as differentially expressed for each comparison.

### Screening hypoxia- or ischemia-associated commonly regulated lncRNAs in humans and mice.

To screen for significantly regulated lncRNAs under ischemic conditions in humans and mice, we looked for common differentially expressed lncRNAs. First, RNA-Seq transcriptomic analysis was performed for normoxia (control) and hypoxia (subject) conditions in mouse SMCs and differentially expressed lncRNAs were obtained (as detailed above). Second, the differentially expressed human lncRNAs (*P* value < 0.05) among healthy aged (HA) and CLTI groups were obtained by analyzing the GSE120642 dataset stored in the GEO database (https://www.ncbi.nlm.nih.gov), which includes skeletal muscle biopsies from 15 healthy controls and 15 patients with CLTI (42). All CLTI patients in this study (42) presented with an ankle-brachial index of less than 0.4 and with ischemic rest pain with or without tissue necrosis or nonhealing ulcers. Third, we performed an intersection of human (mouse homologs) lncRNAs and mouse lncRNAs and ranked the list in mouse based on log_2_ fold change. The lists of human and mouse lncRNAs were obtained from LNCipedia v 5.2 (https://lncipedia.org/) database, and the homologies between human and mouse lncRNAs were retrieved from Mouse Genome Informatics (https://www.informatics.jax.org/) database.

### Statistics.

Statistical analyses were performed using GraphPad Prism version 7.0 (GraphPad Software Inc). Student’s 2-tailed *t* test was used to determine statistical significance between 2 groups. ANOVA with Bonferroni’s test was used to determine differences between more than 2 groups; ordinary 2-way ANOVA with main effects was used to analyze myography data. Data are expressed as mean ± SEM, and results were considered as significantly different using *P* < 0.05.

### Study approval.

Animal protocol (#2016N000182) was approved by the Institutional Animal Care and Use Committee at Brigham and Women’s Hospital, Harvard Medical School and conducted by the National Institutes of Health Guide for Care and Use of Laboratory Animals. This study was approved by the institutional review boards at the University of Florida and the Malcom Randall VA Medical Center (Protocol IRB201801553). All study procedures were carried out according to the Declaration of Helsinki and participants were fully informed about the research and informed consent was obtained. All human patient samples (42) conform to the principles outlined in the Declaration of Helsinki.

### Data availability.

All relevant data are available from the authors. The RNA-seq data are accessible at GEO dataset: (GSE290218). Values for all data points in graphs are reported in the Supporting Data Values file.

## Author contributions

MWF and MZ conceived the hypothesis. MZ, AJ, WWW, EB, VR, JQ, CV, AKP, AKW, MS, and CEA performed experiments. MZ, AJ, WWW, EB, VR, JQ, CV, AKP, AKW, MS, YH, JLG, CEA, TER, WP, and MWF designed or interpreted the results. MZ, AJ, and MWF wrote the manuscript.

## Supplementary Material

Supplemental data

Unedited blot and gel images

Supplemental table 2

Supporting data values

## Version 1 08/28/2025

In-Press Preview

## Version 2 10/15/2025

Electronic publication
