# Supplementary material for: A smooth muscle cell lncRNA controls angiogenesis in chronic limb-threatening ischemia through miR-143-3p/HHIP signaling
Source: J Clin Invest. 2025 Aug 28;135(20):e188559. doi: 10.1172/JCI188559 (PMC12520679; doi:10.1172/JCI188559)
Supplement: Supplemental data [file jci-135-188559-s222.pdf]

Supplementary Figure 1

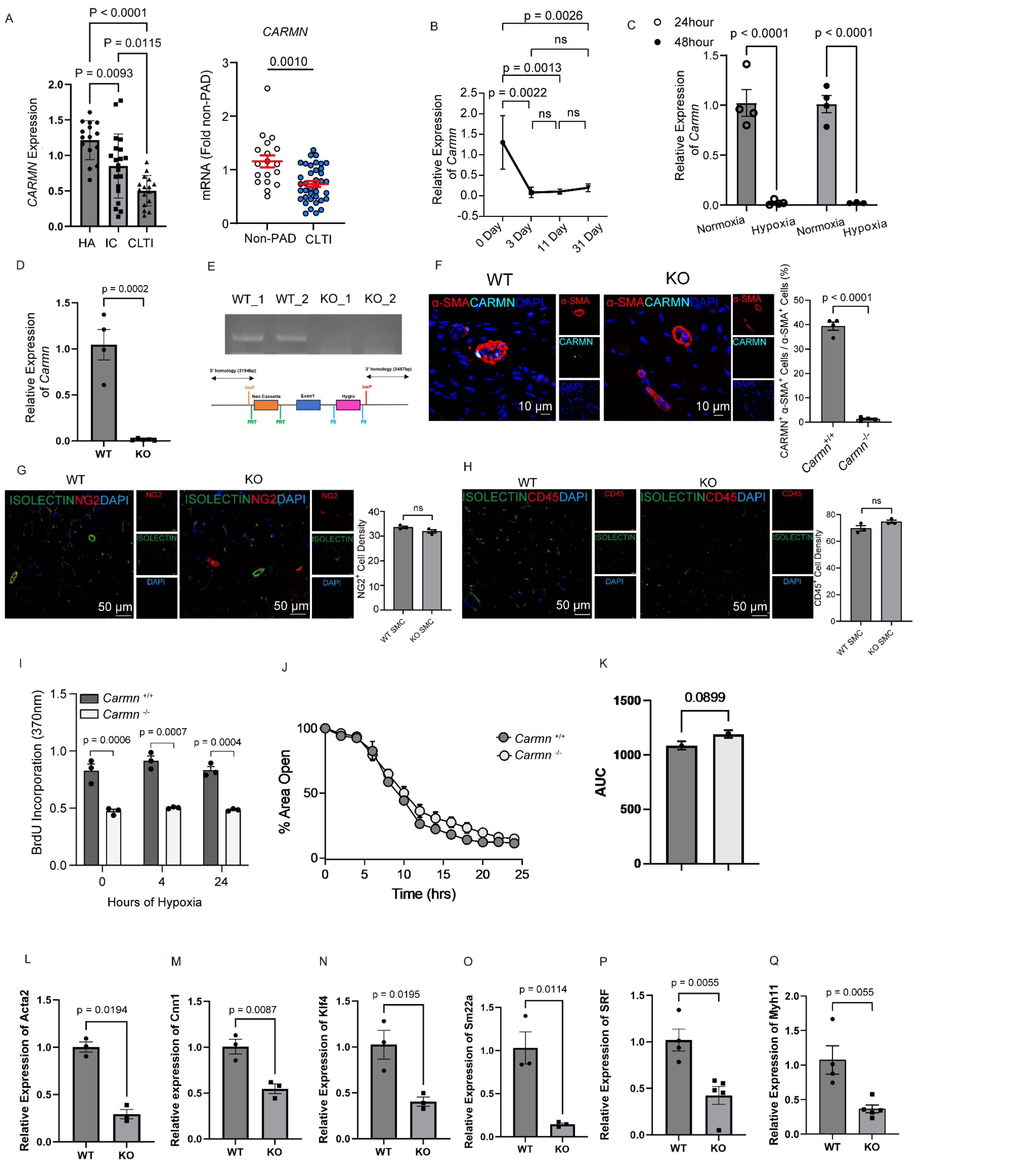

Supplemental Figure 1:

A, Left, Normalized counts of *CARMN* in healthy adults or in patients with intermittent claudication (IC) or critical limb-threatening ischemia from GEO dataset (CLTI) (*n*=13-16) in GEO dataset (GSE120642). Right, the expression of *CARMN* by RT-qPCR in gastrocnemius muscles in the non-PAD control group (n = 18) and CLTI patient group (n=38). B, the relative expression changes of CARMN in gastrocnemius muscle at different time points after femoral artery ligation (FAL) surgery. C, the relative *Carmn* expression in SMCs under normoxic conditions or after 24 or 48 hours of 2% hypoxia. D, the relative expression of *Carmn* between WT and *Carmn* KO SMCs. E, Schematic of the *Carmn* KO mouse construct and genotyping results of *Carmn* WT or KO mice. F, left, the representative image of CARMN co-localizes within the nucleus of α-SMA^{+} SMC within the gastrocnemius muscle harvested from *Carmn* WT and KO groups of mice. Right, and quantification of CARMN^{+}α-SMA^{+} / α-SMA^{+} Cells percentage. G, left, the representative IF image of NG2 (red), isolectin (green), and DAPI (blue); right, the quantification of NG2 pericyte cell density. H, left, the representative IF image of CD45(red), isolectin (green), and DAPI (blue); right, the quantification of CD45 leukocyte cell density. I, BrdU incorporation assay of *Carmn*$^{+/+}$ SMCs and *Carmn*$^{-/-}$ SMCs under different time points of hypoxia. J&K, the image and quantification of the percent open area in the scratch assay between *Carmn*$^{+/+}$ SMCs and *Carmn*$^{-/-}$ SMC, and the area under the curve of scratch assay. L, the relative expression of *Acta2* between WT and CARMN KO SMCs. M, the relative expression of *Cnn1* between WT and CARMN KO SMCs. N, the relative expression of *Klf4* between WT and *Carmn* KO SMCs. O, the relative expression of *Sm22a* between WT and *Carmn* KO SMCs. P, the relative expression of *Srf* between WT and *Carmn* KO SMCs. Q, the relative expression of *Myh11* between WT and *Carmn* KO SMCs. For all panels, error bars represent SEM. These p-values were all determined by unpaired two-tailed Student's *t*-test.

Supplementary Figure 2

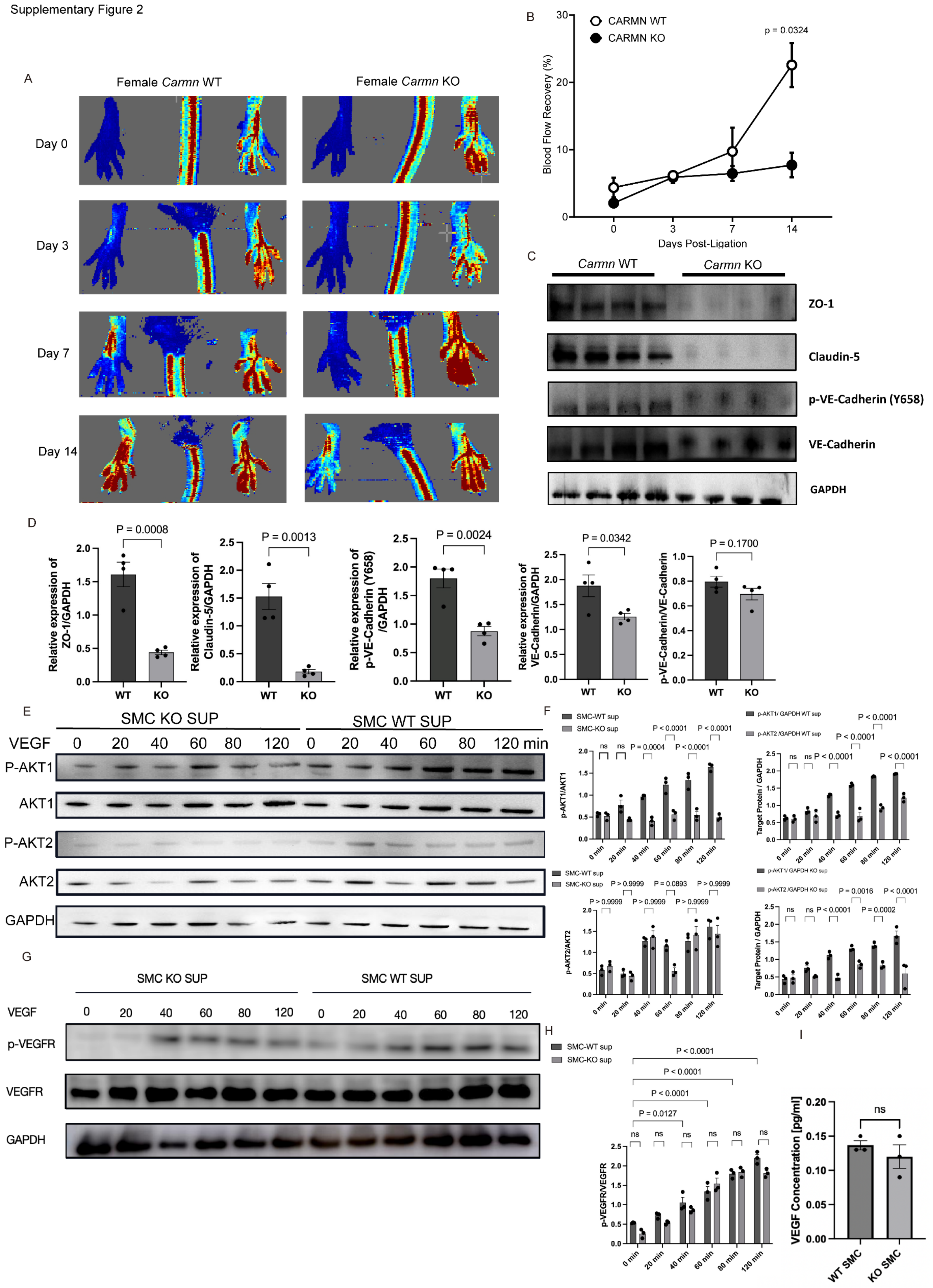

Supplementary Figure 2:

A, Representative Laser Doppler Imaging (LDI) images of hindlimbs immediately after FAL and at different time points of female *Carmn*$^{+/+}$ and female *Carmn*$^{-/-}$ mice. B, Quantification of blood flow (surgical/contralateral limb) by LDI images, normalized to the non-surgery limb between the 2 groups, (*n* = 6). C, Western blot analysis of ZO1, Claudin5, pVE-cadherin-Y658, and VE-cadherin in the gastrocnemius of *Carmn* WT and KO mice after FAL surgery to assess for markers of EC permeability in *vivo*, (*n =4*). D, the quantification of relative expression of ZO1, Claudin5, pVE-cadherin-Y658, and VE-cadherin in the gastrocnemius of *Carmn* WT and KO mice after FAL surgery. E, Representative images of Western blots of the indicated AKT1 and AKT2 proteins in mECs after incubation with WT or KO SMC supernatants. F, quantification of relative expression of the indicated proteins in E. G, Representative images of Western blots of the indicated VEGFR proteins in mECs after incubation with WT or KO SMC supernatants. H, quantification of relative expression of the indicated proteins in G. I, ELISA of VEGF in supernatants of mECs treated with WT or KO supernatants. The *p-value* was determined by one-way ANOVA with Bonferroni post-tests error bars represent SEM (B). The p-value was determined by unpaired two-tailed Student's *t*-test (D, F, H, &I).

Supplementary Figure 3

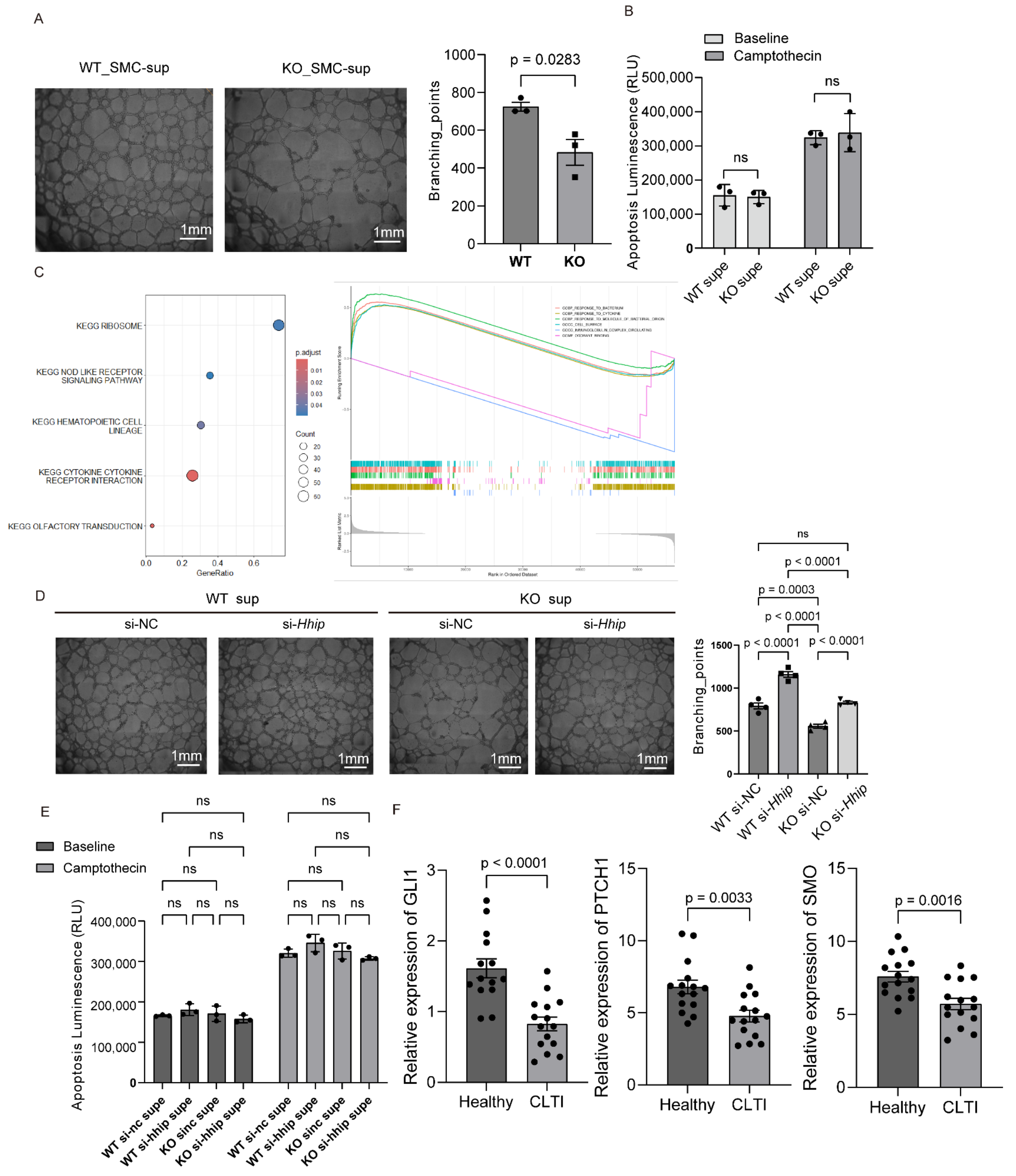

Supplementary Figure 3:

A, representative images (left) of mEC network formation assay incubated with the supernatants collected from WT or *Carmn* KO SMCs and related quantification (right); scale bar = 1mm. The p-value was determined by unpaired two-tailed Student's *t*-test. B, apoptosis assay of mECs incubated with supernatants collected from WT or KO SMCs cultured medium at baseline or with camptothecin treatment. The p-value was determined by unpaired two-tailed Student's *t*-test. C, the bioinformatic pathway analysis of differentially expressed genes between *Carmn* control and *Carmn* knockdown in CASMCs. The data Derived from GSE158972. D, the representative images of network formation assay of mECs incubated with supernatants collected from WT or *Carmn* KO SMCs transfected with si-NC or si-*Hhip* and related quantification. Scale bar = 1mm. E, apoptosis assay of mECs incubated with supernatants collected from WT or *Carmn* KO SMCs transfected with si-NC or si-*Hhip*. For D&E panels, error bars represent SEM. The p-value was determined by one-way ANOVA with Bonferroni post-test. F, Normalized counts of *Gli1, Ptch1,* and *Smo* in healthy adults or in patients with intermittent claudication (IC) or chronic limb-threatening ischemia (CLTI). The p-value was determined by unpaired two-tailed Student's *t*-test.

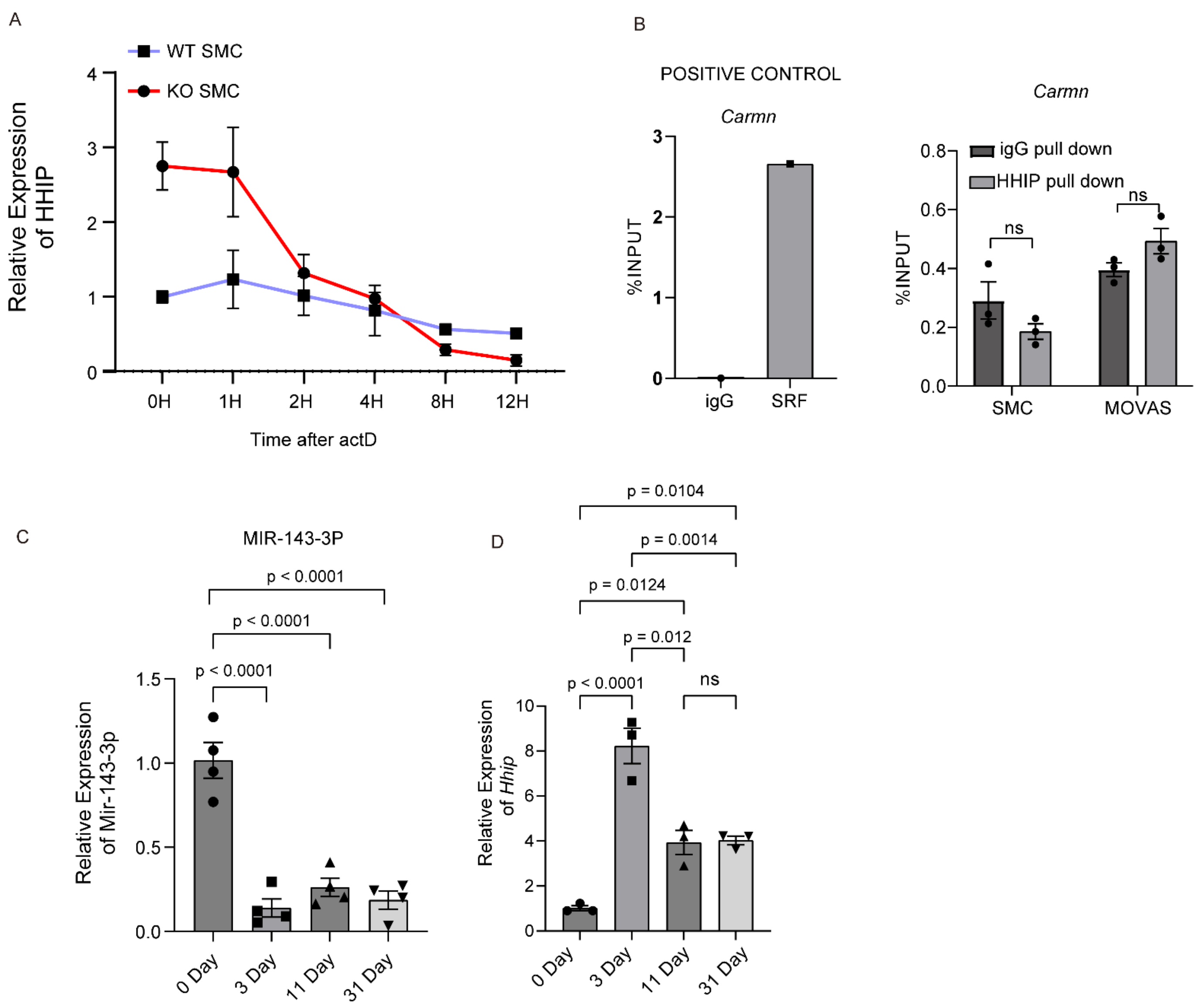

Supplementary Figure 4:

A, the expression changes of Hhip between *Carmn* WT and *Carmn* KO SMCs stimulated with actinomycin D (ActD) at different time points. B, the relative expression of CARMN in IgG and HHIP antibody pull-down groups in HASMC or in SMCs. C, the relative expression of *miR-143-3p* in gastrocnemius muscles of mice after FAL surgery at different time points. D, the relative expression of HHIP in gastrocnemius muscle of mice after FAL surgery at different time points post-surgery. For all panels, error bars represent SEM. The p-value was determined by one-way ANOVA with Bonferroni post-test (A-D).

Supplementary Figure 5

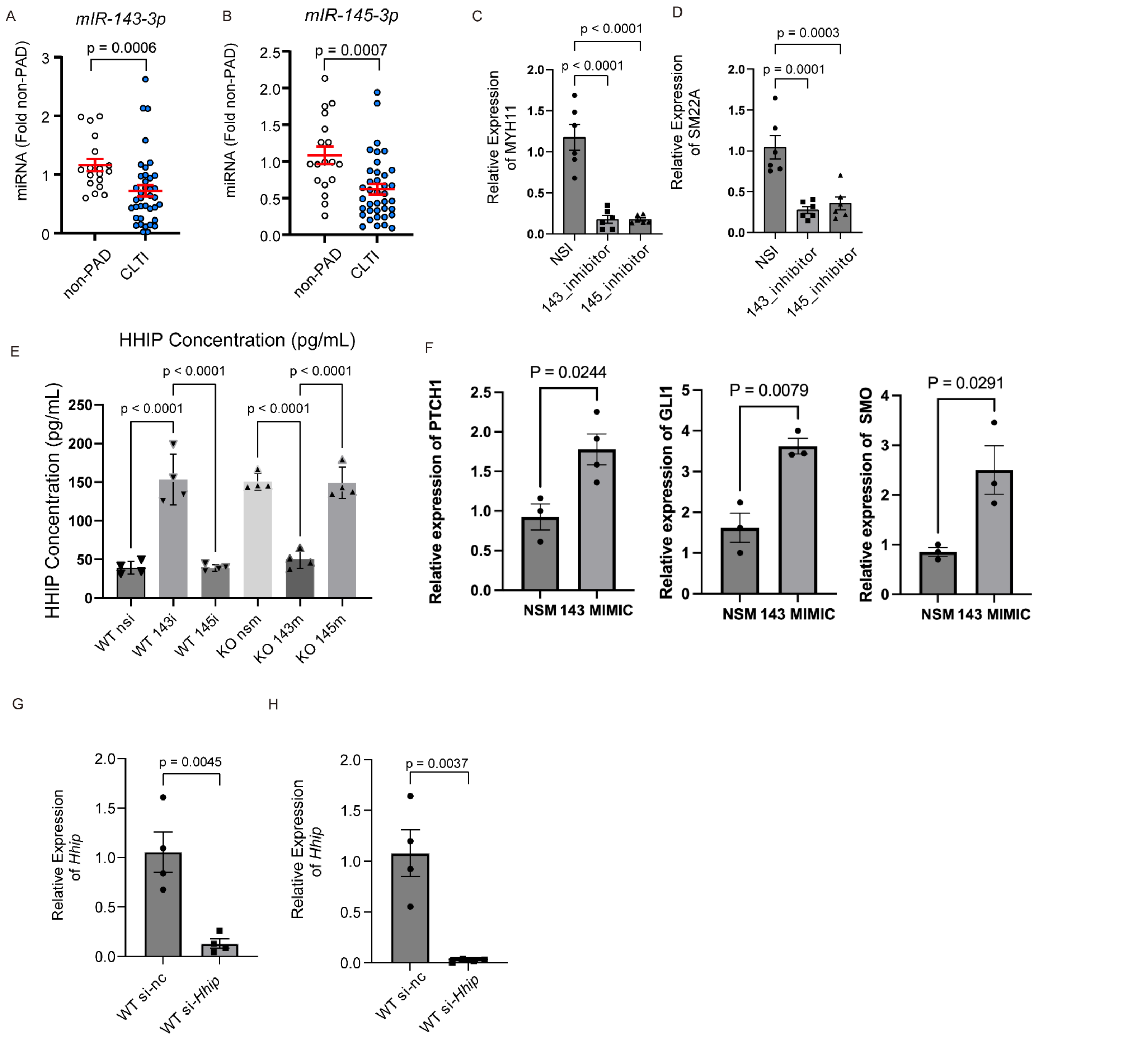

Supplementary Figure 5:

A, the expression of *miR-143-3p* in gastrocnemius muscles in the non-PAD control group (n = 18) and CLTI patient group (n=38). B, the expression of miR-145 in gastrocnemius muscles in the non-PAD control group (n=18) and CLTI patient group (n=38). C, the relative mRNA expression of *Myh11* in WT SMCs transfected with a Non-specific control inhibitor, *miR-143* inhibitor, or *miR-145* inhibitor. D, the relative mRNA expression level of *Sm22a* in WT SMCs transfected with a non-specific control inhibitor, *miR-143* inhibitor, or *miR-145* inhibitor. E, the ELISA assay of HHIP concentration in supernatants collected from WT SMC transfected with a non-specific control inhibitor, *miR-143* inhibitor, or *miR-145* inhibitor; or from KO SMCs transfected with a non-specific control mimic, *miR-143-3p* mimic, or miR-145-5p mimic. F, the expression of Hedgehog genes (*Ptch1*, *Gli1*, and *Smo*) in KO SMCs transfected with NS-m control and *miR-143-3p*. G, the relative expression of *Hhip* in gastrocnemius muscles of ischemic limbs of si-NC injected or si-*Hhip* injected WT mice. H, the relative expression of *Hhip* in gastrocnemius muscles of ischemic limbs of si-NC injected KO mice or si-*Hhip* injected KO mice. For all panels, error bars represent SEM. The p-value was determined by unpaired two-tailed Student's *t*-test (A, B, F, G, &H) or one-way ANOVA with Bonferroni post-test (C, D, &E).

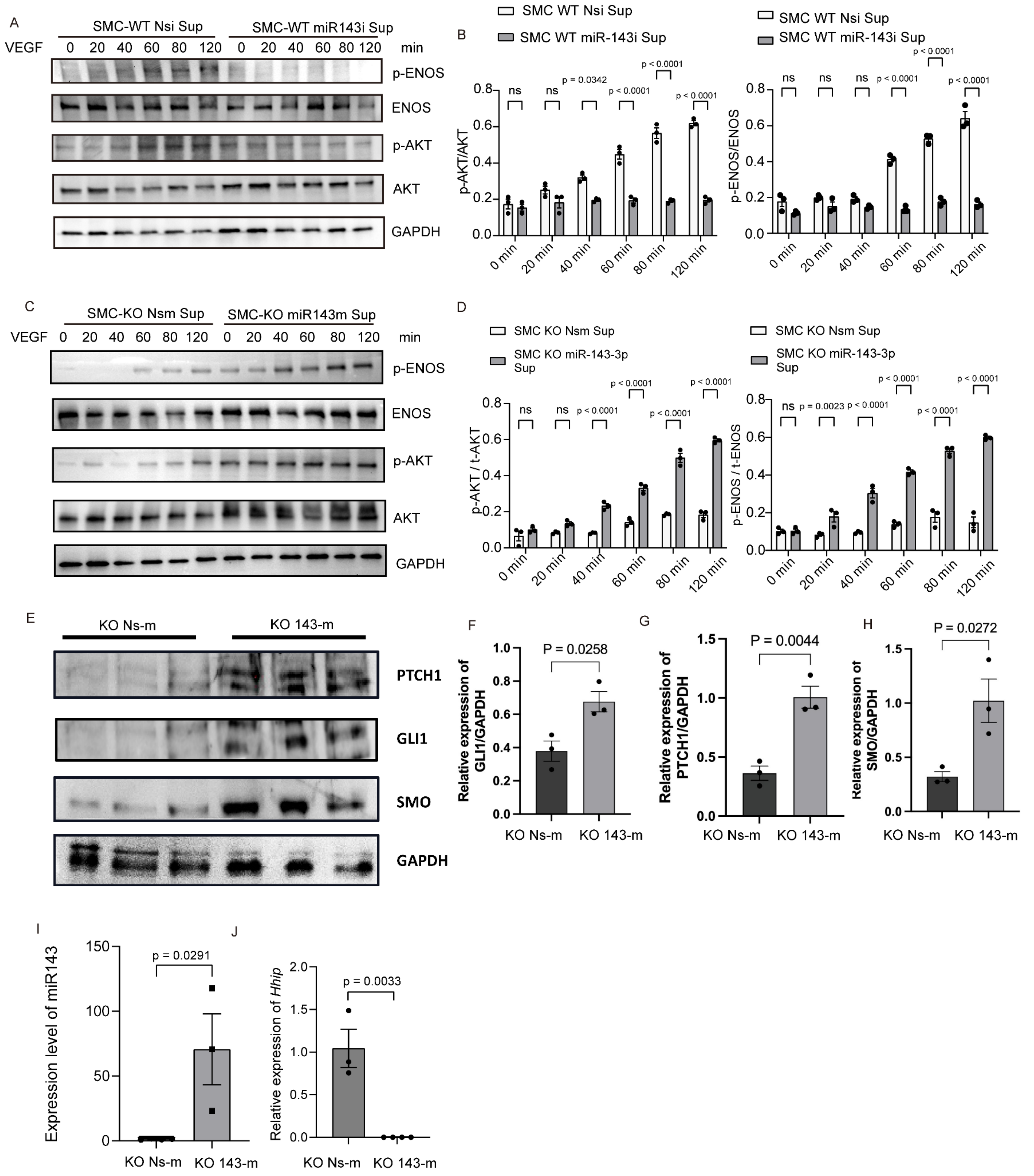

Supplementary Figure 6

A, representative Western blot images of specific proteins of proliferation signaling pathways in mECs treated with supernatants collected from WT SMCs transfected with NSi or *miR-143-3p* inhibitors. B, quantifying the relative expression of pAKT or pENOS of mECs treated supernatants from WT SMCs transfected with NSi or *miR-143-3p* inhibitors. C, representative Western blot images of specific proteins of proliferation signaling pathways in mECs treated with supernatants collected from KO SMCs transfected with NSm or *miR-143-3p* mimics. D, quantifying the relative expression of pAKT or pENOS of mECs treated with supernatants from KO SMCs transfected with NSm or *miR-143-3p* mimics. E, representative Western blot images of the Hedgehog signaling pathway from the gastrocnemius muscle harvested from *Carmn* KO mice after FAL surgery, treated with NS-m control or *miR-143-3p* mimics. F-H, the quantification of relative expression of *Ptch1*, *Gli1*, and *Smo* in E. I, the relative expression of *miR-143-3p* in gastrocnemius muscles of ischemic limbs of NSm (non-specific control) injected KO mice or *miR-143-3p* mimic (143m) injected KO mice. J, the relative expression of *Hhip* in gastrocnemius muscles of ischemic limbs of NSm injected KO mice or *miR-143-3p* mimic injected KO mice. The p-values were determined by unpaired two-tailed Student's *t*-test (B, D, F, G, H, I, &J).

Supplemental Methods

Wire Myography

Isometric tension studies of mesenteric arterioles were performed using wire myography to assess vascular function as previously described.[43] In brief, third-order mesenteric arteries from 10-12 week male WT and CARMN KO mice were isolated and dissected free of perivascular fat. Vessels were maintained in a physiologic salt solution composed of 130 mM NaCl, 4.7 mM KCl, 1.2 mM $MgSO_4$-heptahydrate, 1.2 mM KH_2PO_4, 22 mM $NaHCO_3$, 5.5 mM glucose, and 1.6 mM $CaCl_2$. Two-millimeter segments of vessels were mounted with 40 µm tungsten wires onto a force-transducer-equipped myograph chamber using the DMT 620M multi-myograph system (Danish Myo Technology). Isometric tension recordings were made using LabChart Pro v8 (AD Instruments). Following mounting, vessels were equilibrated at 37°C for 20 minutes. Passive normalization was performed using the DMT Normalization Module in LabChart Pro to determine the optimal vessel diameter and tension to simulate an *in vivo* transmural pressure of 100 mmHg. Following normalization, vessels were exposed to 60 mM KCl prepared in physiologic salt solution to assess vessel integrity. Endothelium-dependent vasorelaxation was then assessed by pre-constricting vessels with 10 µM of phenylephrine; once a stable contraction was achieved, increasing concentrations of acetylcholine were sequentially added while recording vessel tension readings in real-time.

Immunofluorescence

Gastrocnemius muscle was harvested and fixed overnight in 4% paraformaldehyde (PFA) at 4°C. Tissue was washed with PBS three times for 10 minutes each, replacing the PBS each time with fresh solution. Following this step, tissue was dehydrated in a tissue processor, embedded in paraffin, and sectioned. Tissue sections were deparaffinized at 60°C for 1 hour, followed by treatment with Xylene. Sections were then rehydrated in decreasing concentrations of ethanol (100% > 90% > 70%) and distilled water. Antigen retrieval was performed by immersing slides in boiling citrate buffer. Sections were blocked and permeabilized by using 2.5% goat serum (VectorLab, USA) and 0.5% Triton X-100 in 1x PBS for 1 hour at room temperature (RT). Primary antibodies were added to tissue sections at a dilution of 1:250. Sections were incubated at 4°C overnight in a humidified chamber. The next day slides were washed three times with 0.1% PBST and incubated with fluorescently conjugated secondary antibody (1ug/mL) for 1 hour at RT. Slides were washed, mounted with ProLong Gold Antifade with DAPI (catalog # P36935, ThermoFisher, USA), and coverslipped. Nail polish was used as a sealant. Slides were imaged on either a Keyence BZ-x810 epifluorescence microscope or a Zeiss LSM 830 confocal microscope.

ELISA

The supernatants of SMC medium were collected for detecting HHIP expression. Supernatant HHIP levels were measured by ELISA kit (Mouse HHIP ELISA Kit, Cat#orb565922, Biorbyt) according to the manufacturer's instructions.

Luciferase Reporter Assay

HHIP 3'UTR reporter was generated from Genecopoeia (MmiT149045-MT05-01;

NM_001424822.1 wild type clone, and mutated clone). MOVAS cells were co-transfected with 1µg luciferase plasmid vector with 100 nM miR-143-3p mimic or negative control mimic using Lipofectamine 2000 for 24 hours. Luciferase activity was analyzed with Secrete-Pair Dual Luminescence Assay Kit (Genecopoeia, LF031) and standard 96-well plate reader according to the manufacturer's instructions.

RNA Stability Assay
WT or CARMN KO mouse SMCs were seeded in 12-well plates at 70-80% confluence. Next day, SMCs were treated with 5µM Actinomycin-D for 0, 1, 2, 4, 8, or 12-hrs, and RNA was harvested for cDNA synthesis and qPCR analysis. Gene expression is normalized to 0-hr hour baseline group of WT SMCs.

RNA-in situ hybridization (RNA-ISH)
A probe for CARMN was specially developed to detect mouse CARMN (Advanced Cell Diagnostics). Cells or tissues sections were fixed for 2 h in 4% paraformaldehyde and further prepared as described by the manufacturer. *In situ* hybridization was performed using RNAscope 2.5 HD Reagent Kit-Red (Advanced Cell Diagnostics) based on the manufacturer's protocol.

Pathway enrichment analysis
The transcripts commonly differentially expressed (109 upregulated and 182 downregulated) between *in vitro* and *in vivo* groups were considered for the identification of significantly enriched pathways in both groups. Pathway enrichment analysis was performed using QIAGEN-Ingenuity Pathway Analysis (IPA) software (www.ingenuity.com). The Fisher's Exact Test was used to calculate the statistical significance of overlap of differentially expressed transcripts with Canonical Pathways, and the pathways were selected based on statistical significance (p-value<0.05) and enrichment (Z) score. The dynamics of top-significantly enriched pathways in one group (e.g., *in vitro*) was also assessed in the other (e.g., *in vivo*). All analyses were performed in R v4.3.3 (www.r-project.org) statistical environment and the plots were generated with ggplot2 v3.5.1 (ggplot2.tidyverse.org) library.

In vitro functional assays
For EC spheroid sprouting assay, mECs were cultured overnight in hanging drops on nonadherent plastic dishes in Endothelial Cell Medium (Basal Medium (Cell Applications, Cat 210-485) supplemented with Growth Supplement (Cell Applications, Cat 211-GS)) with 0.2% methylcellulose (Sigma-Aldrich) using 1,000 cells/spheroid. Spheroids were embedded in a collagen matrix and incubated for 24 hours with Ctrl or CARMN KO SMC-conditioned media on top (80%) and supplemented with fresh SMC media (20%) and mouse VEGF (RD, Cat#293-VE-010, 50 ng/µl). Number of sprouts and total sprout length of 5-10 spheroids per condition were used for data analysis by using NIH ImageJ software.

For network formation assay, ice-cold matrigel matrix (Cat#354234, Corning, USA) were added into 48-well plates and allowed to polymerize at room temperature (RT) for ~10min. mECs (70,000 cells/well) were resuspended in SMC-conditioned media (supplemented with

20% fresh SMC media) and seeded on polymerized matrigel layer. Cells were imaged live using CytoSMART Omni (Axion Biosystems, USA) imaging system in an incubator set for 37℃ and 5% CO_2. Images were quantified for tube network parameters using IKOSA AI image analyzer (Kolaido, Switzerland).

For cell proliferation evaluation, assays were performed by culturing mECs or SMCs, and then seeding 4,000 cells per well in 96-well plates. Cells were labeled with BrdU labeling reagent and placed in either normoxic conditions (21% O_2) or hypoxic conditions (2-3% O_2) for 8 hours. Cells were subsequently fixed and quantitated using the Cell Proliferation ELISA BrdU Colorimetric Kit according to the manufacturer's instructions (Roche, 11647229001).

Permeability Assay

The endothelial cell permeability assay was performed in a 24-well plate with individual hanging cell culture inserts. First, we pretreated the mECs overnight with supernatant collected from cultivated SMC medium. A diluted cell suspension of 100 µl with ~10^5 cells into the Transwell® insert. Cells became adherent after 30–60 min at 37°C. The mEC medium was mixed with the collected SMC supernatant at 50:50 percent ratio and incubated at 37°C for 24–48 h. A FITC-dextran working solution (150 µl; 10 µg/mL) was added to each insert. The plate was incubated for 20 min at room temperature protected from light. During this time, the FITC-dextran molecules pass through the endothelial monolayer depending on the integrity of the barrier. The plate was read using a fluorescence plate reader with filters appropriate for 485 nm and 535 nm excitation and emission, respectively. The absolute permeability P [cm/s] can be calculated by the following equation:
$P= [C(t) - C(t_0)] \times V/A \times t \times C_0$, the concentration of FITC-dextran in each sample is determined by reference to a FITC-dextran standard curve.

Supplementary Table 1

Patient Characteristics	Non-PAD (N=18)	CLTI (N=38)	*P* value (χ^2 test or *t*-test)
Mean age, y (SD)	68.7 (6.4)	65.6 (8.4)	0.31
Female sex, n (%)	8 (44)	16 (42)	0.88
Non-White, n (%)	5 (27)	10 (26)	0.84
Non-Hispanic, n (%)	17 (94)	36 (94)	0.55
BMI, kg/m^2 (SD)	27.9 (6.3)	27.9 (6.3)	0.97
ABI (SD)	1.01 (0.09)	0.59 (0.27)	1.22E-08
TBI (SD)	0.83 (0.18)	0.35 (0.23)	9.85E-10
Former smoker, n (%)	13 (72)	38 (100)	0.0037
Current smoker, n (%)	3 (16)	6 (16)	0.76
Diabetes type I or II, n (%)	9 (50)	20 (52)	0.92
Hypertension, n (%)	13 (72)	34 (89)	0.21
Hyperlipidemia, n (%)	11 (61)	33 (86)	0.0652
Coronary artery disease, n (%)	8 (44)	24 (63)	0.27
Aspirin, n (%)	10 (55)	30 (78)	0.13
ACE inhibitor, n (%)	5 (27)	18 (47)	0.27
Angiotensin receptor blocker, n (%)	3 (16)	8 (21)	1.00
Statin, n (%)	14 (77)	35 (92)	0.27
Cilostazol, n (%)	0 (0)	11 (28)	0.0288
Beta Blocker, n (%)	11 (61)	26 (68)	0.80
Anticoagulant, n (%)	2 (11)	14 (36)	0.0943
Antiplatelet, n (%)	4 (22)	12 (32)	0.68
