# Supplementary material for: A smooth muscle cell lncRNA controls angiogenesis in chronic limb-threatening ischemia through miR-143-3p/HHIP signaling
Source: J Clin Invest. 2025 Aug 28;135(20):e188559. doi: 10.1172/JCI188559 (PMC12520679; doi:10.1172/JCI188559)

For Figure 2E

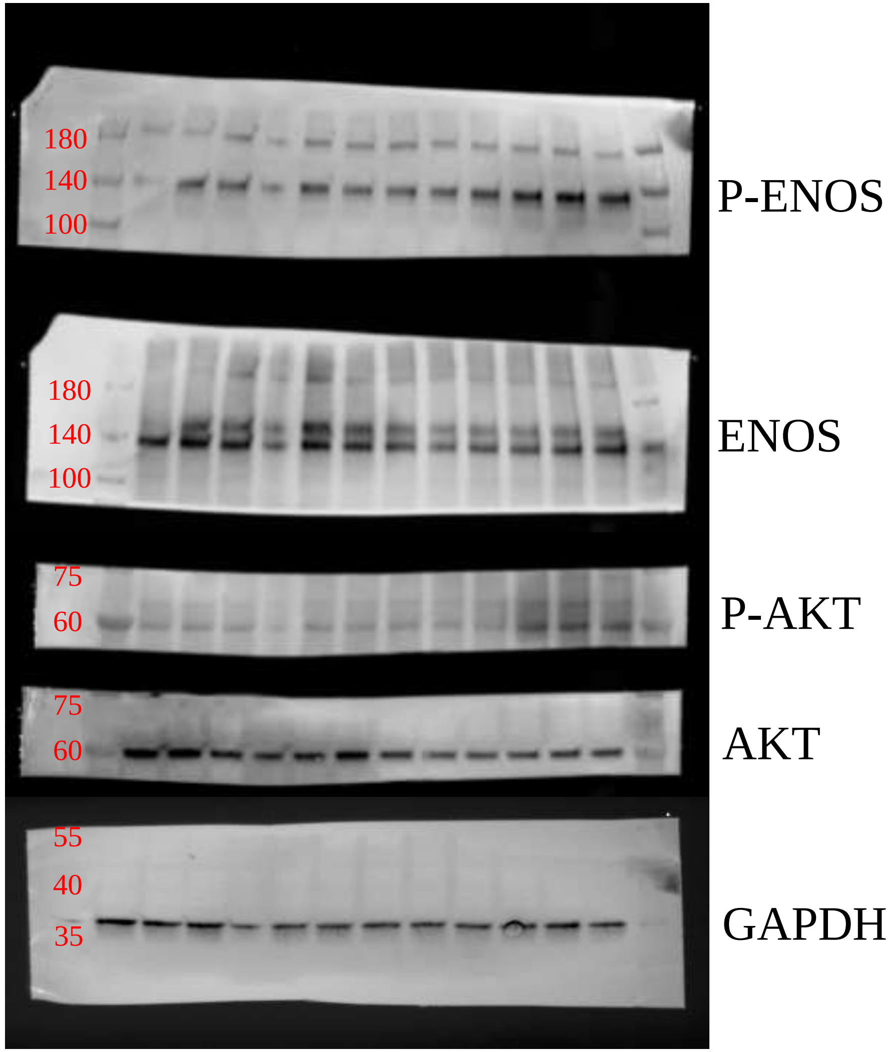

For Figure 4C

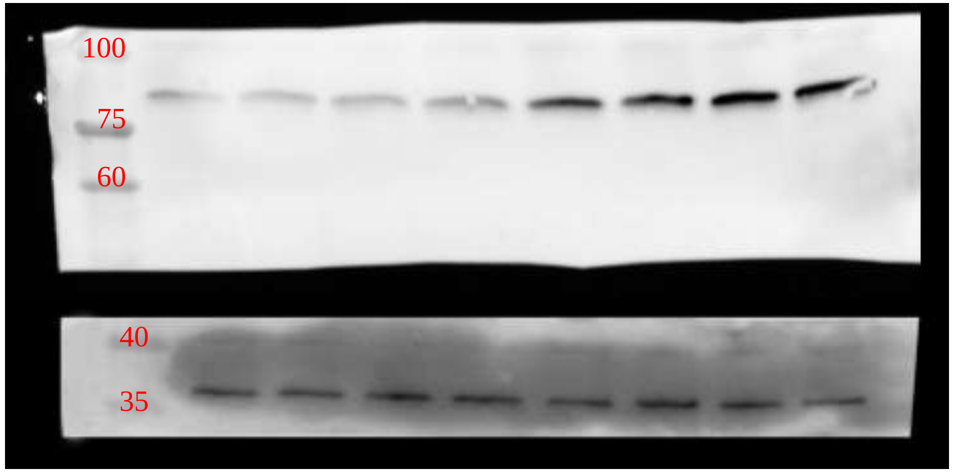

HHIP

GAPDH

For Figure 5B

Upper

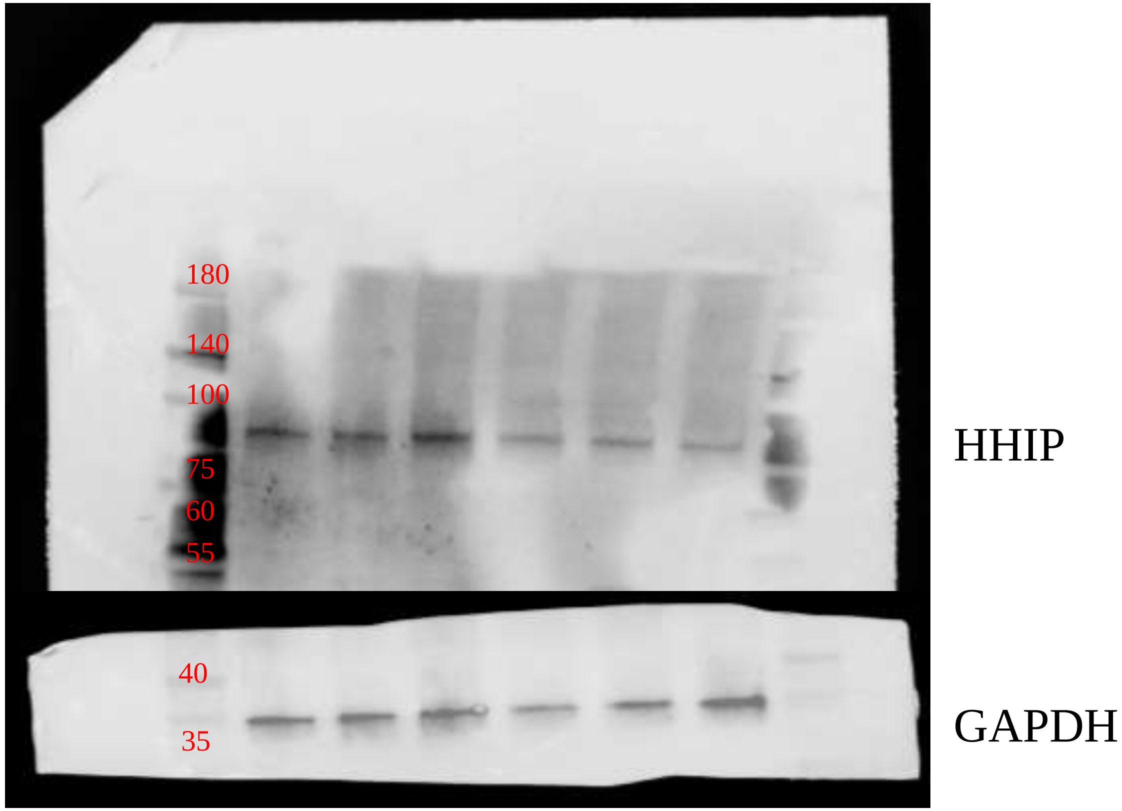

Lower

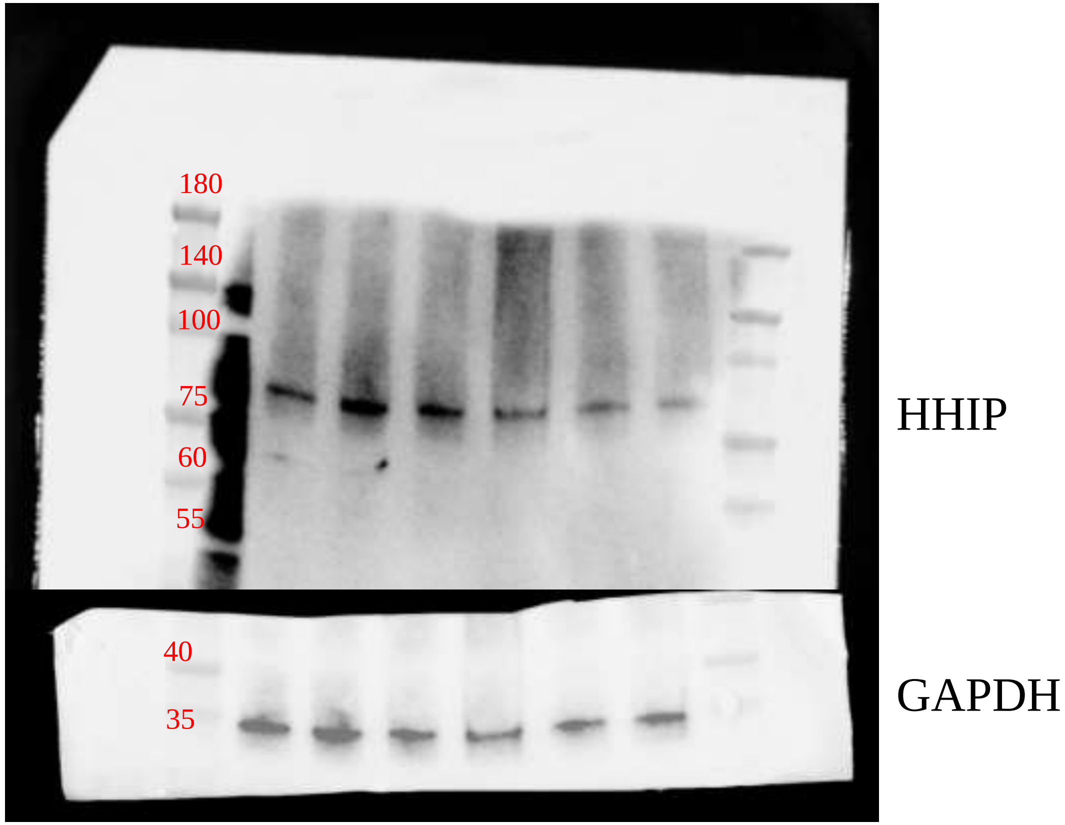

For Figure 5F

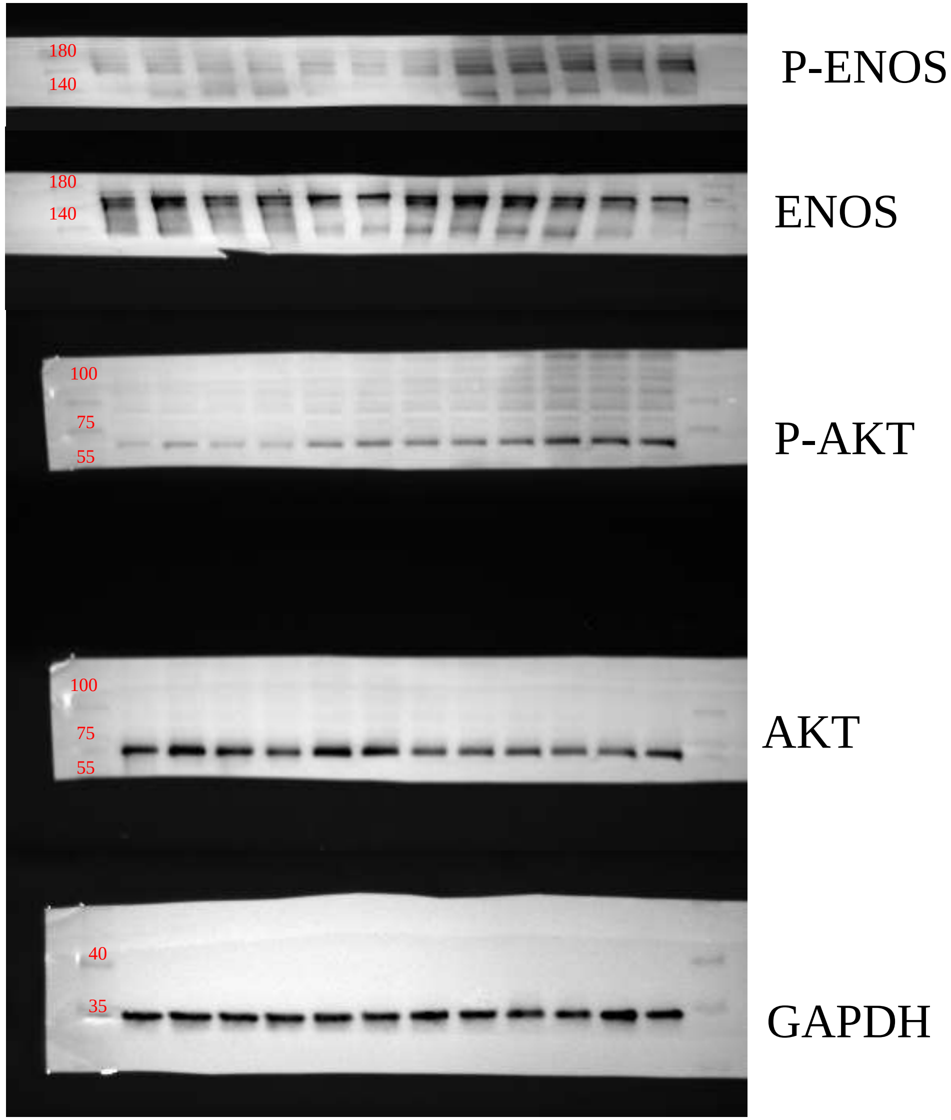

For Figure 5I

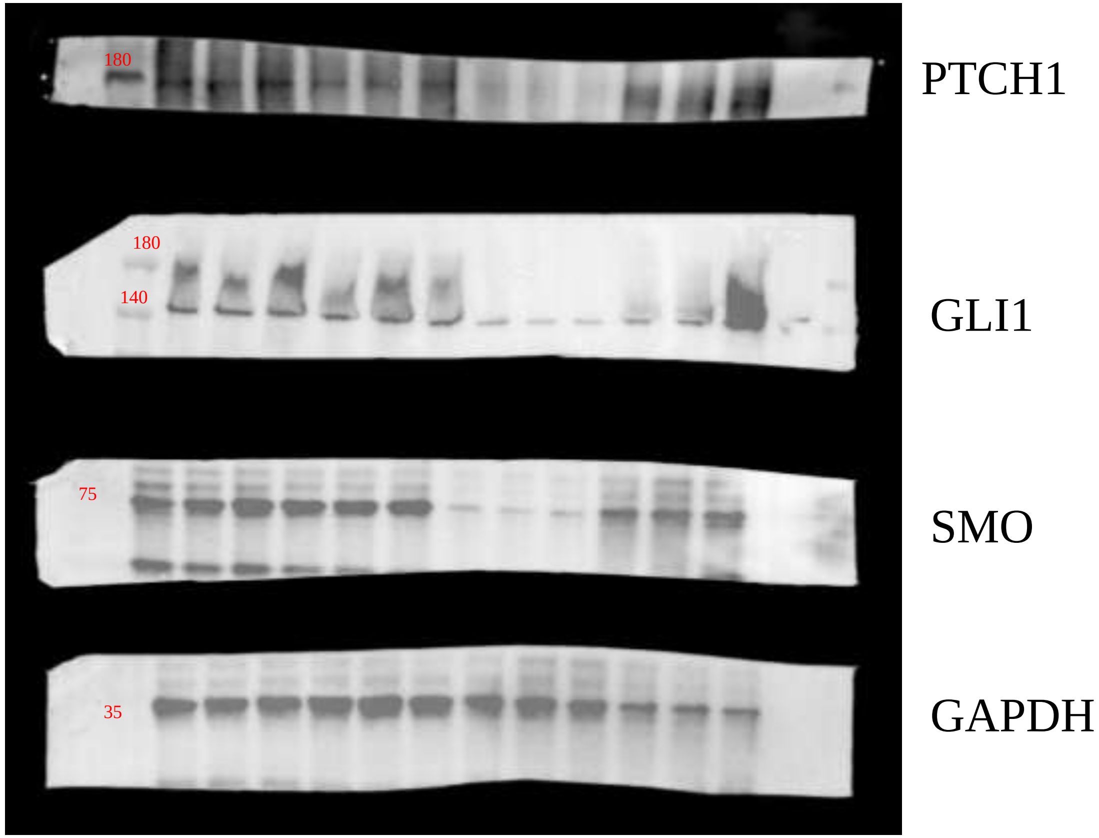

For Figure 6D

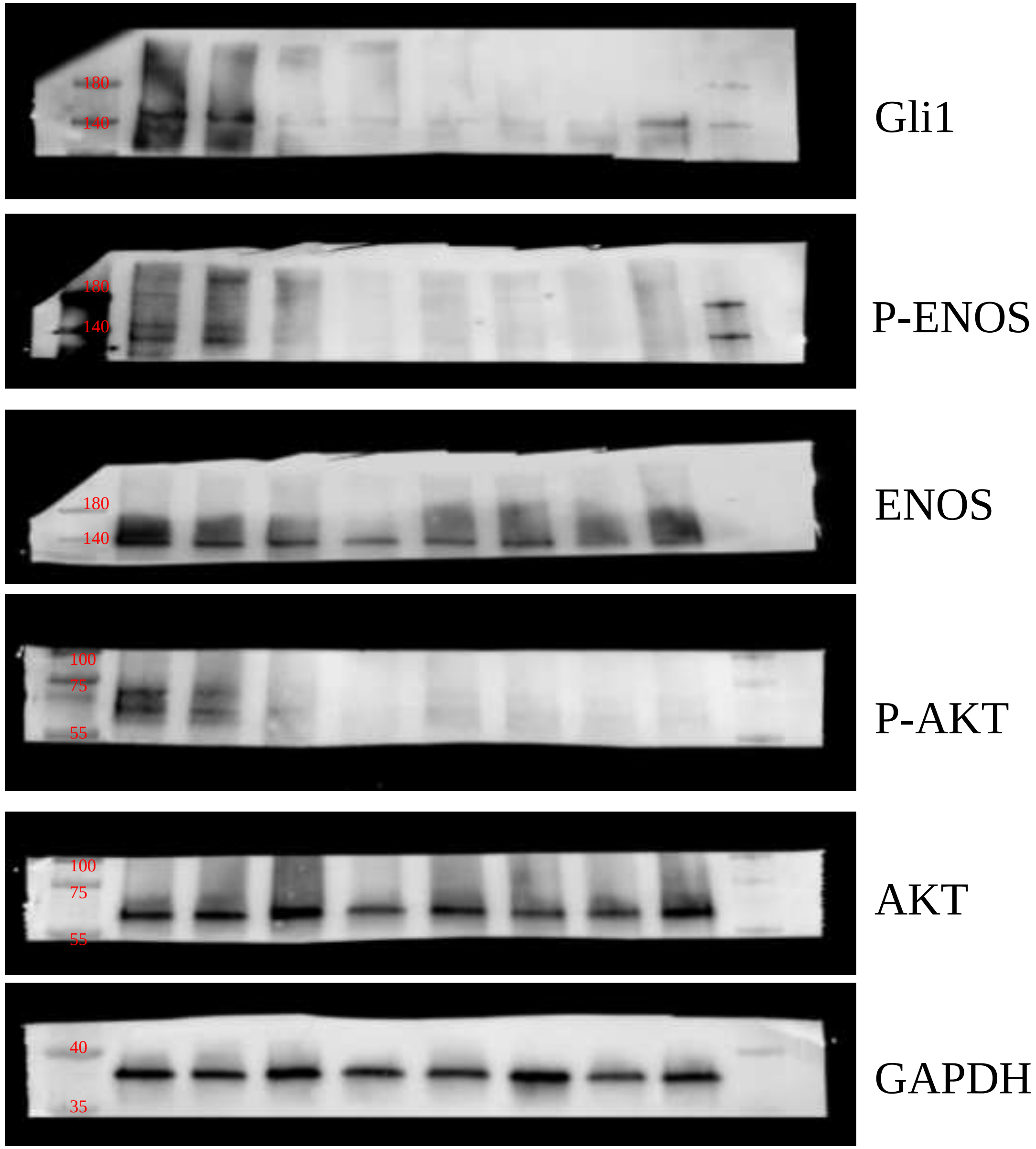

For Figure 7E

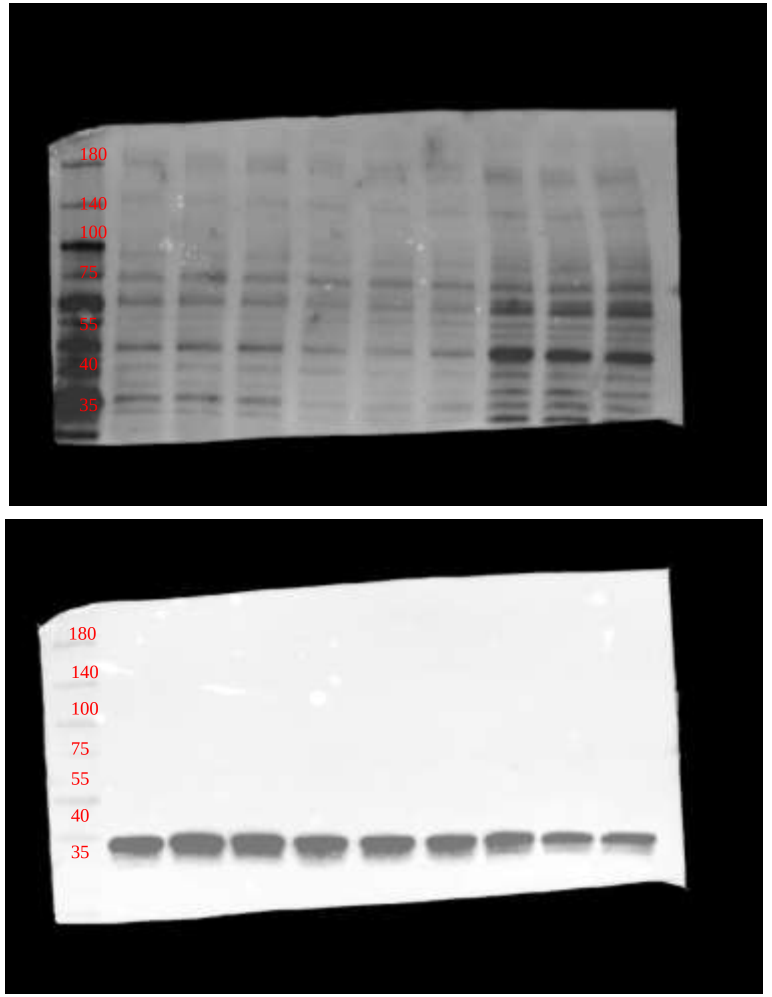

HHIP

GAPDH

For Figure 7N

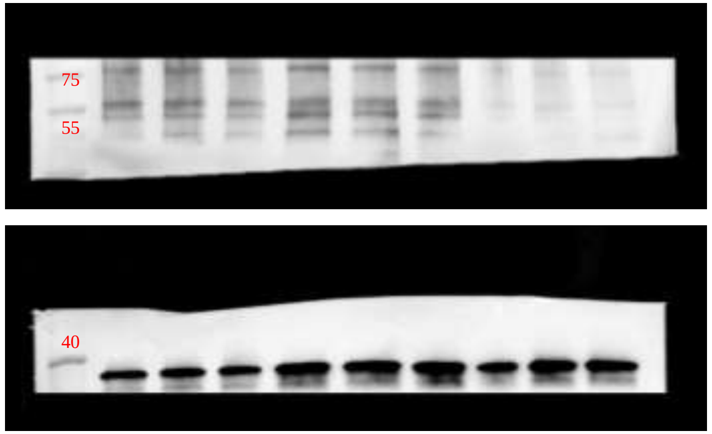

HHIP

GAPDH

For Supplementary Figure 6A

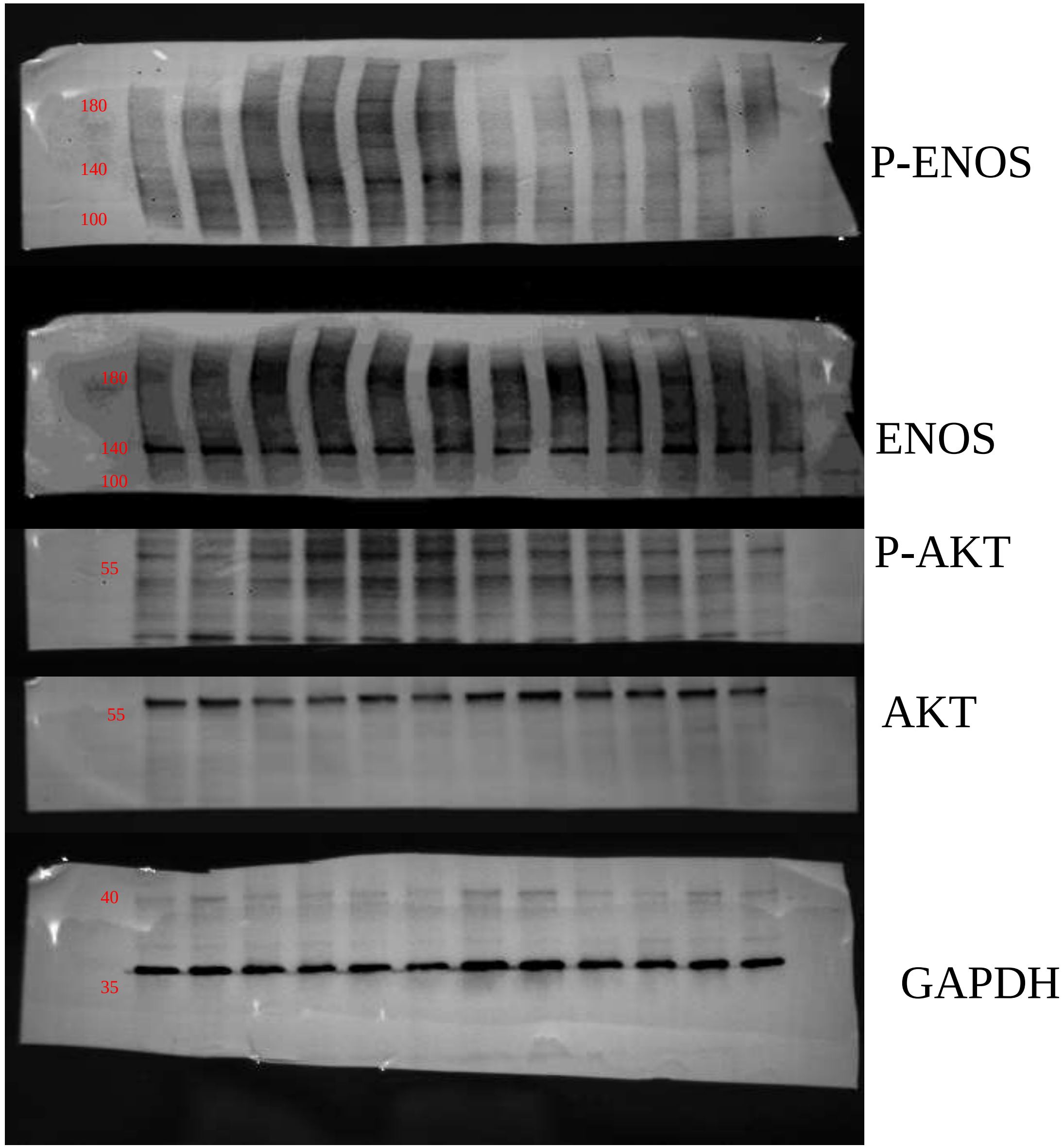

For Supplementary Figure 6B

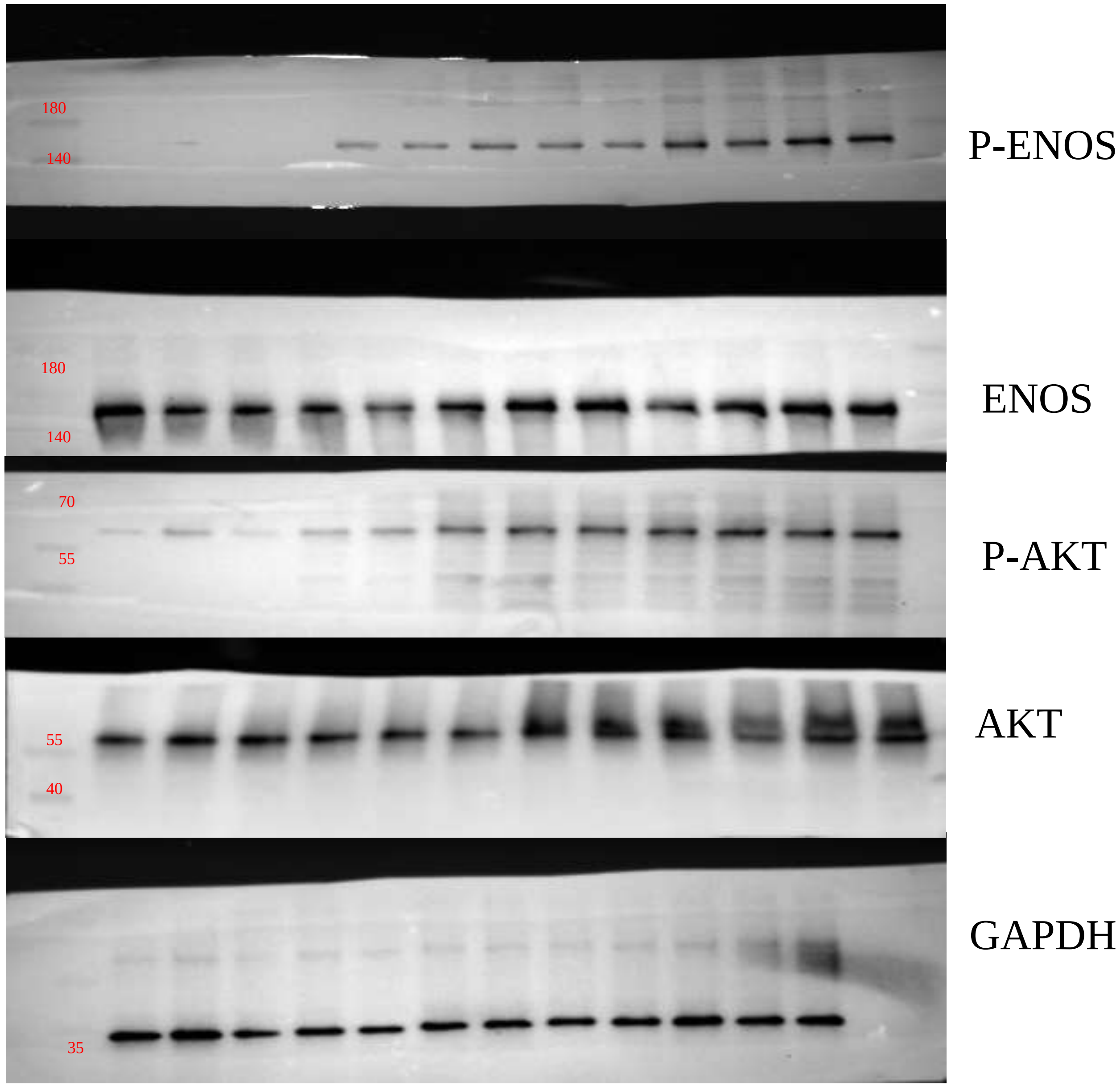

For Supplementary Figure 7A

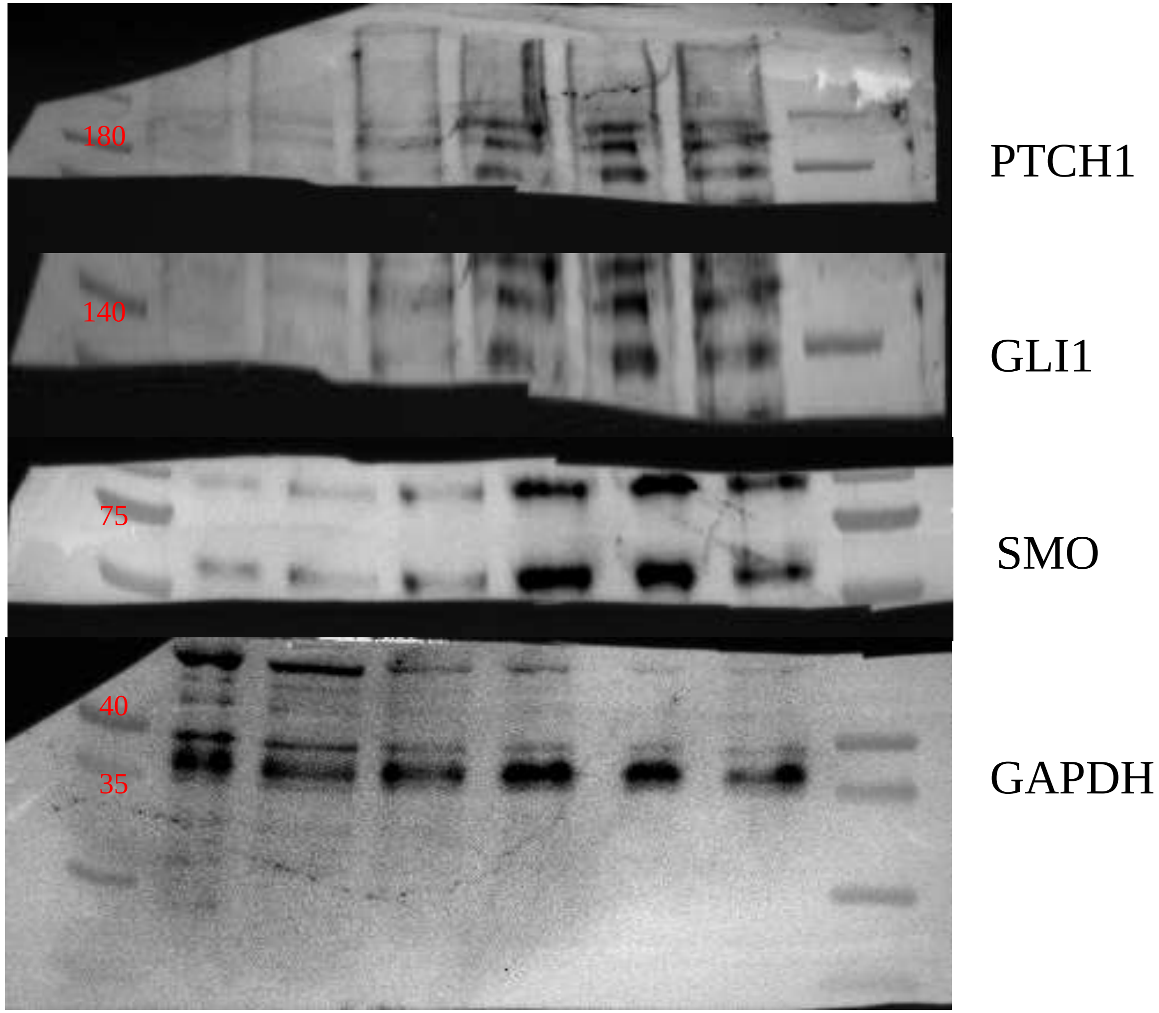

For Supplementary Figure 7B

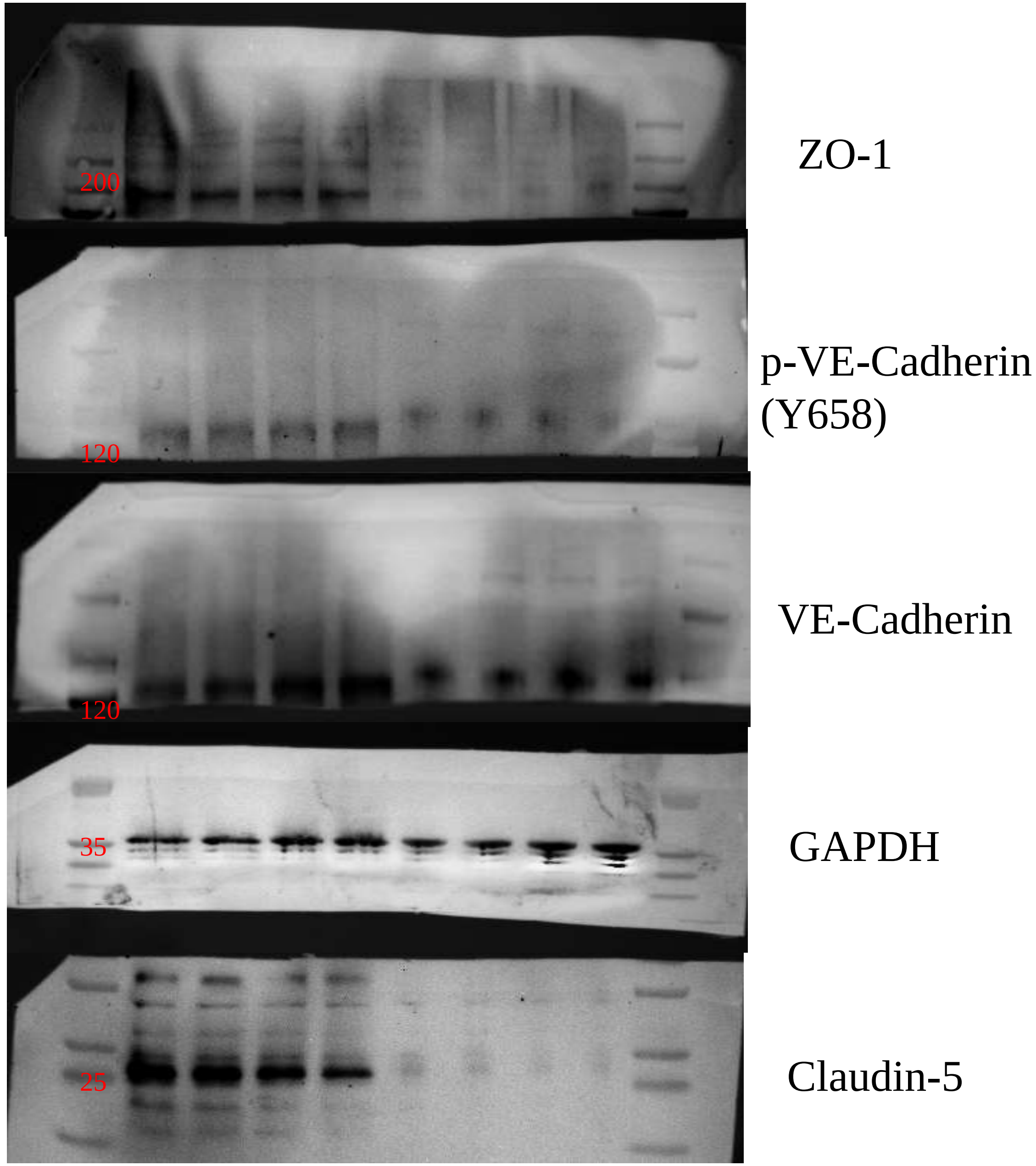

For Supplementary Figure 2E&F

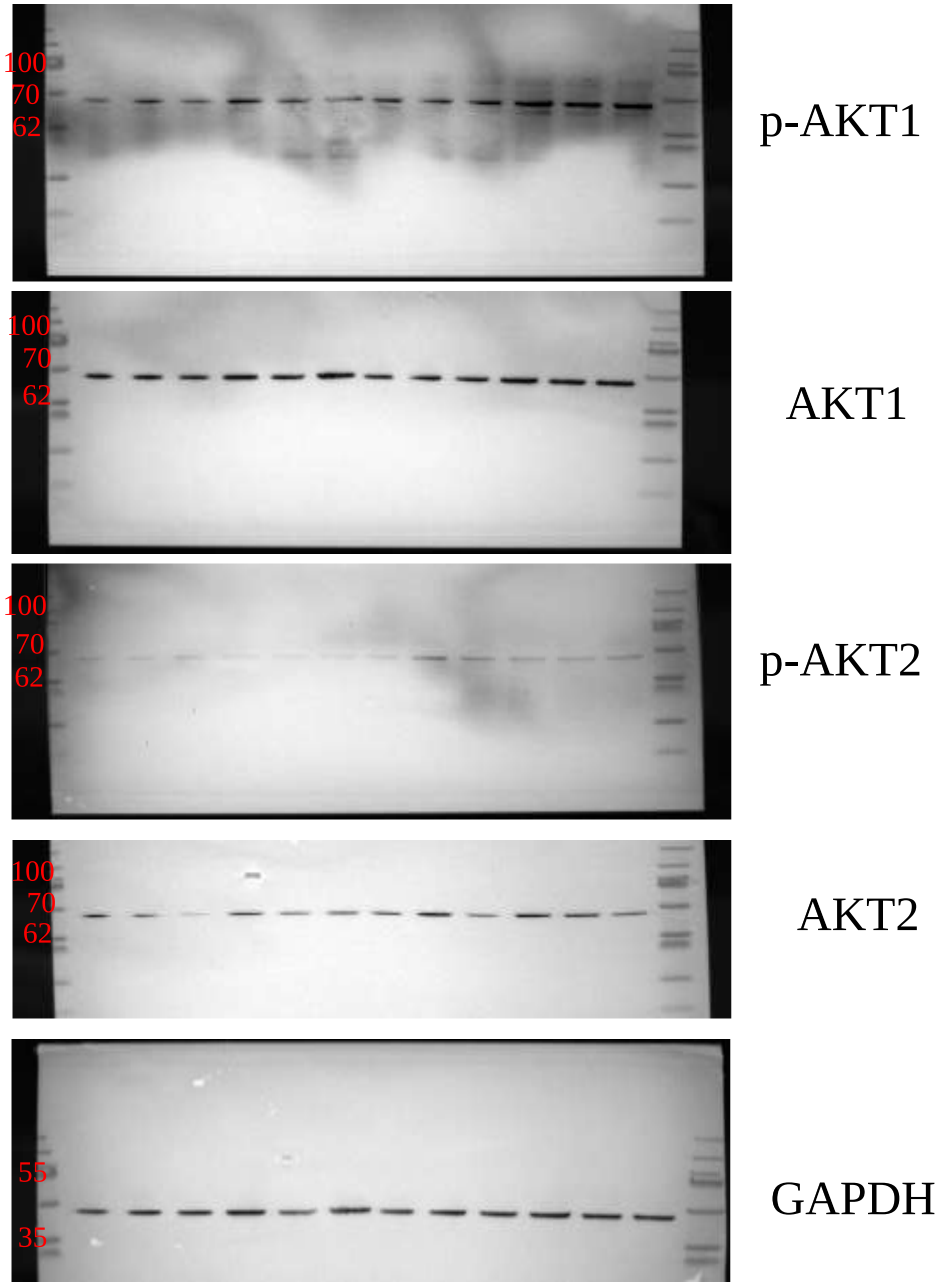

For Supplementary Figure 2G-H

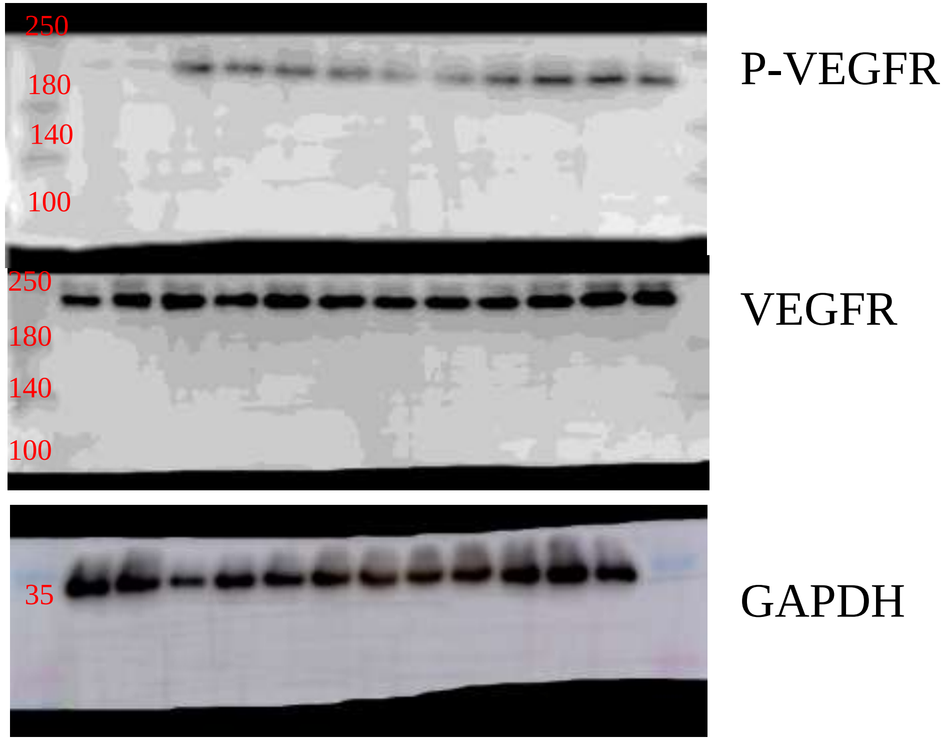

Supplement: Unedited blot and gel images [file jci-135-188559-s223.pdf]
